# GPRC5A is a potential oncogene in pancreatic ductal adenocarcinoma cells that is upregulated by gemcitabine with help from HuR

**DOI:** 10.1038/cddis.2016.169

**Published:** 2016-07-14

**Authors:** H Zhou, A G Telonis, Y Jing, N L Xia, L Biederman, M Jimbo, F Blanco, E Londin, J R Brody, I Rigoutsos

**Affiliations:** 1 Computational Medicine Center, Sidney Kimmel Medical College, Thomas Jefferson University, 1020 Locust Street Philadelphia, PA 19107, USA; 2 Department of Neuroscience and The Farber Institute for Neuroscience, Thomas Jefferson University, 900 Walnut Street, Philadelphia, PA 19107, USA; 3 Department of Pathology, Anatomy and Cell Biology, Sidney Kimmel Medical College, Thomas Jefferson University, 1020 Locust Street, Philadelphia, PA 19107, USA; 4 Department of Surgery, The Jefferson Biliary and Related Cancer Center, Sidney Kimmel Medical College, Thomas Jefferson University, 1025 Walnut Street, Philadelphia, PA 19107, USA

## Abstract

*GPRC5A* is an orphan G-protein coupled receptor with an intriguing dual behavior, acting as an oncogene in some cancers and as a tumor suppressor in other cancers. In the pancreatic cancer context, very little is known about GPRC5A. By analyzing messenger RNA (mRNA) expression data from 675 human cancer cell lines and 10 609 samples from The Cancer Genome Atlas (TCGA) we found that GPRC5A's abundance in pancreatic cancer is highest (cell lines) or second highest (TCGA) among all tissues and cancer types. Further analyses of an independent set of 252 pancreatic normal and cancer samples showed GPRC5A mRNA to be more than twofold upregulated in primary tumor samples compared with normal pancreas (*P*-value<10^−5^), and even further upregulated in pancreatic cancer metastases to various organs (*P*-value=0.0021). Immunostaining of 208 cores (103 samples) of a tissue microarray showed generally low expression of *GPRC5A* protein in normal pancreatic ductal cells; on the other hand, in primary and metastatic samples, *GPRC5A* protein levels were dramatically increased in pancreatic ductal cells. *In vitro* studies of multiple pancreatic cancer cell lines showed that an increase in *GPRC5A* protein levels promoted pancreatic cancer cell growth and migration. Unexpectedly, when we treated pancreatic cancer cell lines with gemcitabine (2′,2′-difluorodeoxycytidine), we observed an *increase* in *GPRC5A* protein abundance. On the other hand, when we knocked down GPRC5A we sensitized pancreatic cancer cells to gemcitabine. Through further experimentation we showed that the monotonic increase in *GPRC5A* protein levels that we observe for the first 18 h following gemcitabine treatment results from interactions between GPRC5A's mRNA and the RNA-binding protein HuR, which is an established key mediator of gemcitabine's efficacy in cancer cells. As we discovered, the interaction between GPRC5A and HuR is mediated by at least one HuR-binding site in GPRC5A's mRNA. Our findings indicate that GPRC5A is part of a complex molecular axis that involves gemcitabine and HuR, and, possibly, other genes. Further work is warranted before it can be established unequivocally that GPRC5A is an oncogene in the pancreatic cancer context.

Pancreatic cancer is lethal and the fourth leading cause of cancer deaths in the United States with a 5-year overall survival rate of 6.7%.^1^ In 2014, more than 46 000 people were diagnosed with pancreatic cancer in the United States. Pancreatic ductal adenocarcinoma (PDAC) currently accounts for most of the diagnosed cases. Despite great efforts and very significant progress in elucidating the molecular events of pancreatic tumorigenesis, many of the details remain unknown. The disease's idiosyncratic attributes (e.g. cellular and molecular heterogeneity, extensive peritumoral stroma and unknown drug resistance mechanisms) have made it difficult to target both established (e.g. K-ras mutations) and more recently uncovered PDAC-specific molecular events.^2^ To date, the majority of PDAC studies have focused on elucidating the impact of genetic mutations, the role of proteins, and the role of microRNAs (miRNAs) and their interactions with messenger RNAs (mRNAs).^1, 3^

Recent research reports have suggested that the G-protein-coupled receptor, class C, group 5, member A or *GPRC5A* for short, may play important roles in a variety of settings.^4^ *GPRC5A* was first discovered in 1998 and became known initially as retinoic acid-induced gene 3 (*RAI3*) or retinoic acid-induced gene 1 (*RAIG1*).^5^ Very early experiments with multiple breast cancer cell lines linked the gene to the malignancy whereas siRNA knockdown in a single pancreatic cell line showed the induction of morphological changes.^6^ The available data to date suggest that the protein is very abundant in normal lung, normal liver, normal kidney, normal cerebellum, breast cancer, pancreatic cancer, etc.^4, 5^ The protein localizes primarily at the plasma membrane (see: The Human Protein Atlas, http://www.proteinatlas.org) and at cytoplasmic organelles, including the perinuclear vesicle, endoplasmic reticulum and the Golgi apparatus whereas, extracellularly, it has been identified in vesicular exosomes.^4, 5, 7, 8, 9, 10^ GPRC5A has so far been shown to be dysregulated in many different human cancers as well as in other diseases.^4, 6, 10, 11, 12, 13, 14, 15, 16, 17, 18, 19, 20, 21, 22, 23, 24, 25, 26^ However, an increase in its abundance is not always associated with the cancer state: for example, in lung cancer GPRC5A levels are lower than in adjacent normal tissues;^24^ on the other hand, in several breast cancers GPRC5A levels are higher than in adjacent normal tissues.^16, 21^

Mechanistically, GPRC5A was shown to interact with both non-coding RNAs (ncRNAs) and proteins.^4, 6, 22, 23, 24, 25, 26, 27, 28, 29, 30, 31, 32, 33, 34, 35, 36, 37, 38, 39^ Post-translationally, GPRC5A protein can be phosphorylated by cyclin-dependent kinases in the cells,^40, 41, 42, 43^ which suggests a role in cell cycle progression. The protein can also be N-glycosylated and ubiquitinated.^4, 27, 28, 33, 34, 36, 37^ Dysregulation of GPRC5A was also shown to result in abnormal activation of NF-*κ*B signaling and STAT3 signaling.^44^ Recently, it was also shown that in breast cancer GPRC5A may be involved in DNA repair in cooperation with BRCA1.^22^ From a post-transcriptional regulation standpoint, however, very little is known.

ELAV-like protein 1, also known as ‘human antigen R' or HuR, is an RNA-binding protein that is encoded by the *ELAVL1* gene.^45, 46^ The HuR protein comprises three RNA-binding domains^47, 48^ and has been found to bind preferentially AU-rich motifs in the 3′UTR of mRNA transcripts, thereby increasing their stability.^49, 50^ In terms of location, HuR is primarily found in the nucleus but translocates to the cytoplasm under the control of endogenous and exogenous factors.^51^ HuR is known to regulate post-transcriptionally multiple genes and non-coding RNAs^52, 53, 54^ and to play key roles in human malignancies.^55^ Specifically in the pancreatic cancer context, HuR has been shown to regulate deoxycytidine kinase (dCK), an enzyme that activates gemcitabine (2′,2′-difluorodeoxycytidine), thereby contributing, at least in part, to gemcitabine's efficacy in these cancer cells.^56^

In this report, we provide evidence that supports the hypothesis that *GPRC5A* acts as an oncogene in the pancreatic cancer context. Through a series of experiments with multiple pancreatic cancer cell lines, we examine the impact of GPRC5A overexpression on cell growth, colony formation ability and migration. In addition, we examine the role of the RNA-binding protein HuR, a key mediator of gemcitabine efficacy, in post-transcriptionally regulating GPRC5A and assess the ability of gemcitabine to modulate the abundance of GPRC5A in pancreatic cells. Lastly, we evaluate the impact of GPRC5A knockdown on the cancer cells' sensitivity to gemcitabine.

## Results

### Gene expression analyses of numerous cell lines and human samples show that GPRC5A mRNA levels in pancreatic cancer are among the highest and even further elevated in metastases

**Figure 1:**
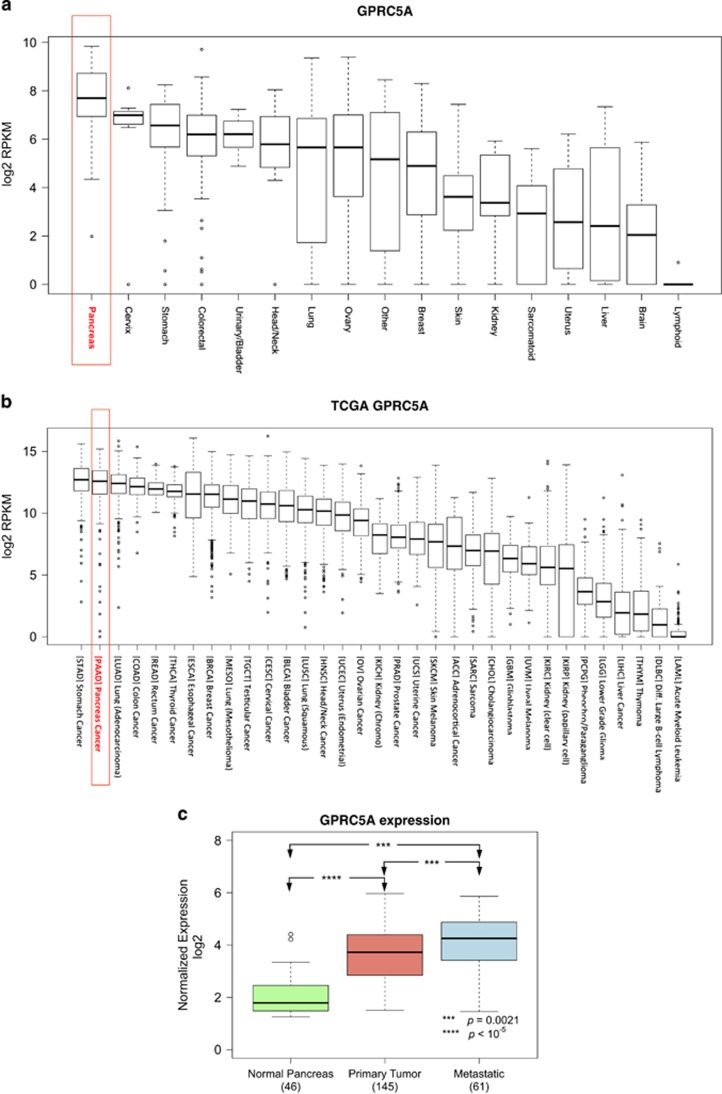
GPRC5A is highly expressed in pancreatic cancer cell lines and pancreatic cancer tissue. (**a**) RNA-seq datasets showing GPRC5A mRNA expression in different cancer cell lines. (**b**) RNA-seq data from TCGA showing GPRC5A mRNA expression in different cancers. (**c**) GPRC5A mRNA expression in normal pancreas, primary PDAC samples and metastases

First, we analyzed publicly available RNA-seq data from 675 human cancer cell lines representing 17 human tissues.^57^ As can be seen in Figure 1a, GPRC5A mRNA is widely expressed across different tissues and cell line types. Its average expression is highest in pancreatic cancer cell lines. Then, we extended our analysis to the 10 609 samples of the TCGA repository, which represent 33 different cancer types. As Figure 1b shows, GPRC5A mRNA is present abundantly across multiple cancers and exhibits the second highest average abundance in pancreatic ductal adenocarcinoma (PAAD). Having established that across all cancers GPRC5A is very abundant in pancreatic cancer, we analyzed previously reported microarray data^2^ (GEO accession number: GSE71729) and examined GPRC5A expression in normal pancreatic tissue (*n*=46), primary pancreatic tumors (*n*=145) and metastatic tumors (*n*=61) using the R statistical computing environment.^58^ Figure 1c shows that compared with normal pancreas, GPRC5A is upregulated in primary tumors (*P*-value <10^−5^) and in pancreatic metastases (*P*-value=0.0021). As can be seen from the same figure, metastatic tumors had an even higher average expression of GPRC5A compared with primary tumors.

### Immunohistochemical analyses show a dramatic increase of *GPRC5A* protein levels in primary PDAC samples and in several metastases

**Figure 2:**
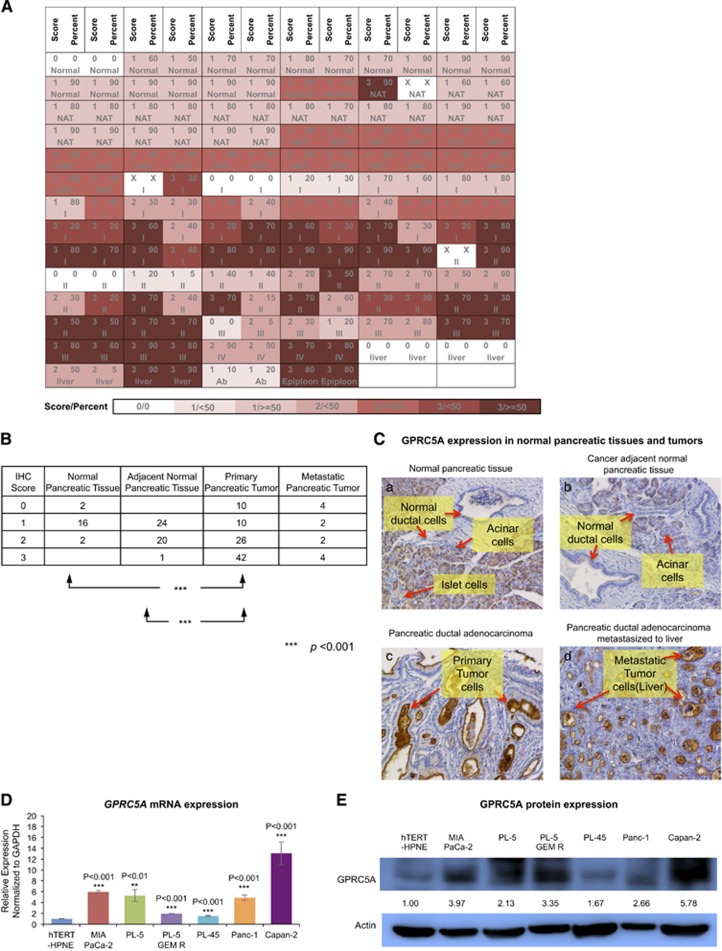
GPRC5A is upregulated in pancreatic ductal adenocarcinoma at both the mRNA level and the protein level. (**a**) Score table of TMA staining of 20 cores of normal pancreatic tissues, 42 cores of adjacent normal pancreatic tissues, 90 cores of primary pancreatic ductal adenocarcinoma and 12 cores of metastatic pancreatic ductal adenocarcinoma. (**b**) Summary of (**a**). (**c**) Examples of immunohistochemistry staining of GPRC5A in normal pancreatic tissues, primary PDAC samples and metastases. (**d**) GPRC5A mRNA expression in different pancreatic cell lines. (**e**) GPRC5A protein expression in different pancreatic cell lines. All numerical data are mean±S.D. *P<0.05, **P<0.01, ***P<0.001, *n*=3 or as indicated. Ab, pancreatic ductal tumor metastases to abdominal cavity; Epiploon, pancreatic ductal tumor metastases to epiploon; Liver, pancreatic ductal tumor metastases to liver; NAT, adjacent normal tissue; Normal, normal pancreatic tissue; I,II,III, IV, pancreatic ductal cancer stages

Having demonstrated with public data that GPRC5A mRNA is upregulated in both primary PDAC and metastases compared with normal pancreas, we sought to investigate whether *GPRC5A* protein levels changed in a concomitant manner. To this end, we stained 208 samples from 103 cases contained in a tissue microarray (PA2081a; US Biomax Inc, Rockville, MD, USA) with the same GPRC5A antibody (HPA007928; Sigma-Aldrich, St. Louis, MO, USA) that is used by the Human Protein Atlas (http://www.proteinatlas.org). We found that generally in both normal pancreatic tissue and adjacent normal pancreatic tissue from tumor, there was low to medium expression of *GPRC5A* protein in pancreatic ductal cells; however, in primary PDAC samples, *GPRC5A* protein levels increased dramatically (Figure 2a and b). The high levels of *GPRC5A* protein in PDAC tumor cells persisted even when the cells metastasized to other organs like liver (Figure 2c). Supplementary Figure S1 shows additional examples of the stained samples from the tissue microarray.

### GPRC5A mRNA and protein levels are high in multiple PDAC cell lines

We examined both GPRC5A mRNA and protein expression levels in six pancreatic cancer cell lines and in the normal pancreatic epithelial cell line hTERT-HPNE that is derived from the pancreas duct. As shown in Figure 2d, the level of GPRC5A mRNA in the six pancreatic cancer cell lines is statistically significantly higher compared with hTERT-HPNE. In concordance with the mRNA levels, *GPRC5A* protein levels are also increased in the six PDAC cell lines compared with hTERT-HPNE (Figure 2e and Supplementary Figure S2A). Our quantitative polymerase chain reaction (qPCR) results are mirrored by the GPRC5A mRNA abundance in 38 pancreatic cell lines as gauged by next generation sequencing^57^ (Figure 1a).

### Overexpression of GPRC5A promotes colony formation and migration in normal epithelial pancreatic cells

**Figure 3:**
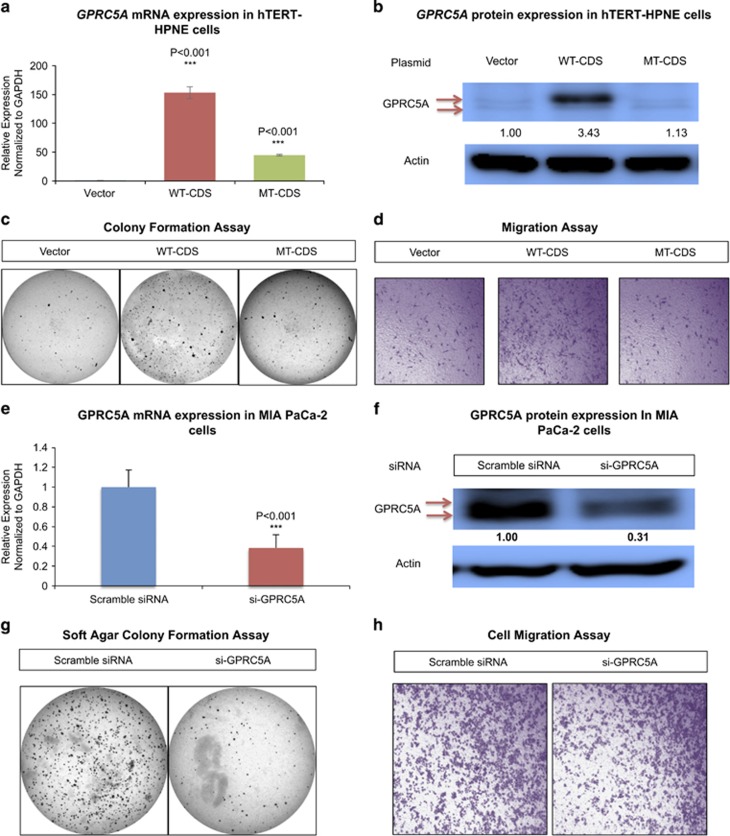
Ectopic expression of GPRC5A protein has an impact on pancreatic cells' colony formation ability and migration ability. (**a**) GPRC5A mRNA expression in hTERT-HPNE cells after overexpression of GPRC5A WT-CDS or GPRC5A MT-CDS. (**b**) GPRC5A protein expression in hTERT-HPNE cells after overexpression of GPRC5A WT-CDS or GPRC5A MT-CDS. (**c**) Soft agar colony formation assay performed with hTERT-HPNE cells after overexpression of GPRC5A WT-CDS or GPRC5A MT-CDS. (**d**) Cell migration assay performed with hTERT-HPNE cells after overexpression of GPRC5A WT-CDS or GPRC5A MT-CDS. (**e**) GPRC5A mRNA expression in MIA PaCa-2 cells after treatment with siRNA. (**f**) GPRC5A protein expression in MIA PaCa-2 cells after treatment with siRNA. (**g**) Soft agar colony formation assay performed with MIA PaCa-2 cells after siRNA treatment. (**h**) Cell migration assay performed with MIA PaCa-2 cells after siRNA treatment. All numerical data are mean±S.D. *P<0.05, **P<0.01, ***P<0.001, *n*=3

We overexpressed GPRC5A protein in hTERT-HPNE cells and examined its impact on the cells' ability to form colonies and to migrate. As controls, we alternately transfected hTERT-HPNE cells with a GPRC5A overexpression construct whose start codon was mutated, or, with the empty vector (Figures 3a and b and Supplementary Figure S2B). As demonstrated by the results shown in Figures 3c and d and in Supplementary Figures S2C and D, an increase in the abundance of *GPRC5A* protein promotes the ability of hTERT-HPNE cells to form colonies and to migrate. On the other hand, increasing the abundance of GPRC5A's mRNA has no such effect.

### Inhibition of GPRC5A impairs colony formation and migration in PDAC cell lines

We designed an siRNA, si-GPRC5A (Figures 3e and f and Supplementary Figure S2E), and used it to examine whether inhibition of GPRC5A in PDAC cells could hinder colony formation and cell migration. Si-GPRC5A (the targeted sequence is shown in Supplementary Table S1) specifically targets GPRC5A's 3′UTR. As our results in Figures 3g and h and in Supplementary Figures S2F and G show, inhibition of GPRC5A in MIA PaCa-2 cells impaired the cells' colony formation in soft agar as well as reduced their migration ability. Moreover, when we inhibited GPRC5A in the pancreatic cancer cell lines Panc-1 and Capan-2 we could inhibit the cells' migration ability (Supplementary Figures S3A–D). Interestingly, we also found that inhibition of GPRC5A could hinder colony formation even in the normal epithelial pancreatic cell line hTERT-HPNE (Supplementary Figures S3E–H).

### Inhibition of *GPRC5A* in PDAC cells diminishes cell survival following gemcitabine treatment

**Figure 4:**
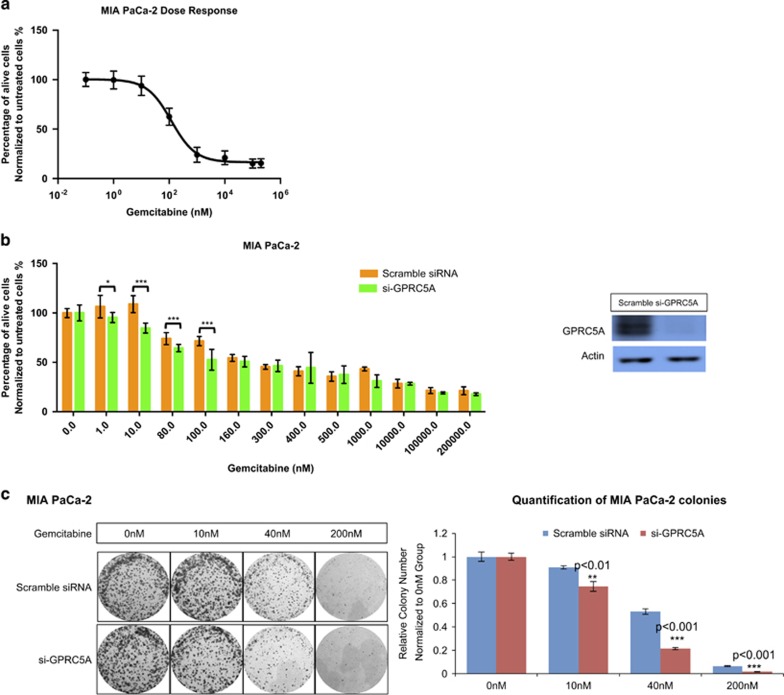
Knockdown of GPRC5A enhances pancreatic cancer cells' death in face of gemcitabine treatment. (**a**) MIA PaCa-2 cells' response to different concentrations of gemcitabine treatment. (**b**) Knockdown of GPRC5A in MIA PaCa-2 cells enhanced cells' death following gemcitabine treatment. (**c**) Knockdown of GPRC5A in MIA PaCa-2 cells reduced cells' colony formation ability when treated with gemcitabine. All numerical data are mean±S.D. * *P*<0.05, ** *P*<0.01, *** *P*<0.001, *n*=3

In light of the fact that GPRC5A knockdown reduced the ability of MIA PaCa-2 cells to form colonies and to migrate, we considered whether modulating the abundance of GPRC5A in parallel to gemcitabine treatment could affect the cells' sensitivity to the drug. We used si-GPRC5A to inhibit GPRC5A in MIA PaCa-2 cells, and then treated the cells with increasing concentrations of gemcitabine: we found that GPRC5A knockdown resulted in a decrease of cell survival and reduction of the cells' colony formation ability in the presence of gemcitabine at concentrations around EC_50_ (Figure 4). Experimentation with two more pancreatic cancer cell lines (Panc-1, PL-5) showed similar improvements in their gemcitabine sensitivity following GPRC5A knockdown (Supplementary Figures S4A–F). We also experimented with a gemcitabine-resistant PL-5 cell line (PL-5-GEM R): in this case, modulation of GPRC5A's abundance had an effect on the cells' response only when treated with high concentrations of gemcitabine (Supplementary Figures S4G–H). We also tested the effect of GPRC5A inhibition on MIA PaCa-2 cells' response to six more chemotherapy drugs (Supplementary Figure S5). With the exception of Trichostatin A, inhibition of GPRC5A in MIA PaCa-2 cells did not show significant effects on the cells' response to these drugs.

### Gemcitabine treatment increases *GPRC5A* protein expression in MIA PaCa-2 cells through post-transcriptional events

**Figure 5:**
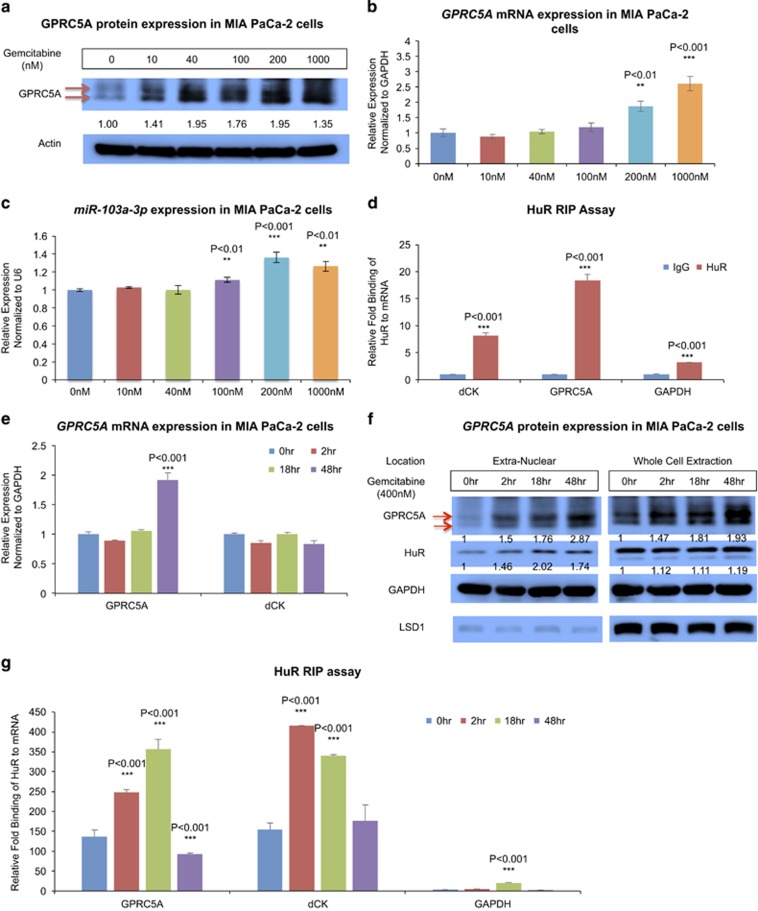
Gemcitabine treatment enhances GPRC5A expression in MIA PaCa-2 cells due to HuR in the early stage. (**a**) GPRC5A protein expression in MIA PaCa-2 cells after 48 h of treatment with gemcitabine at different concentrations. (**b**) GPRC5A mRNA expression in MIA PaCa-2 cells after 48 h treatment of gemcitabine in different concentrations. (**c**) miR-103a-3p expression in MIA PaCa-2 cells after 48 h of treatment with gemcitabine at different concentrations. (**d**) HuR RIP assay in MIA PaCa-2 cells. (**e**) GPRC5A mRNA expression in MIA PaCa-2 cells after 400 nM gemcitabine treatment for 0, 2, 18 and 48 h. (**f**) GPRC5A protein and HuR protein levels in the cytoplasm or the whole-cell lysate of MIA PaCa-2 cells after 400 nM gemcitabine treatment for 0, 2, 18 and 48 h. (**g**) HuR RIP assay performed in MIA PaCa-2 cells after 400 nM gemcitabine treatment for 0, 2, 18 and 48 h. All numerical data are mean±S.D. *P<0.05, **P<0.01, ***P<0.001, *n*=3

We next treated MIA PaCa-2 cells with increasing concentrations of gemcitabine and examined whether treatment affected GPRC5A protein and mRNA levels. We found that upon gemcitabine exposure for 48 h, *GPRC5A* protein expression increased monotonically with increasing gemcitabine concentrations eventually reaching a plateau at ~100 nM followed by a drop at 1 *μ*M (Figure 5a and Supplementary Figure S6A). Quantitation of GPRC5A mRNA levels revealed again two distinct regimes but they were different compared with those of the protein levels: there was little increase of mRNA abundance at lower gemcitabine concentrations followed by dramatically increases in mRNA abundance at higher concentrations (Figure 5b). Similar result was also shown in PL-5 cells (Supplementary Figures S6B–D).

This complementary behavior suggested the existence of post-transcriptional regulatory events that are induced by gemcitabine. The absence of changes in mRNA abundance in the regime of concentrations where protein expression increased suggested the possibility of transcript stabilization. On the other hand, the marked increase of GPRC5A mRNA at higher gemcitabine concentrations suggested a different mode of modulation that could result from transcriptional as well as post-transcriptional changes.

In recent work, we showed that miR-103a-3p interacts with the 5′-UTR of GPRC5A mRNA, thereby decreasing the abundance of both mRNA and protein.^59^ To examine the possibility of an miRNA-mediated post-transcriptional contribution to the observed mRNA abundance changes across different gemcitabine concentrations, we quantitated the corresponding levels of miR-103a-3p. As Figure 5c shows, miR-103a-3p levels *increase* at a high gemcitabine concentration (200–1000 nM), yet remains largely constant at lower concentrations (10–100 nM). There is lack of concordance between the miRNA and the mRNA and protein level changes shown in Figures 5a and c at a low concentration of gemcitabine treatment. The findings suggest that much of the observed modulation of GPRC5A protein abundance at low gemcitabine concentrations is *not* due to miR-103a-3p.

There are several possibilities that might underlie the apparent mRNA stabilization suggested by the increase of protein abundance at lower gemcitabine concentrations (Figure 5a), an increase that occurs without evident changes of mRNA at the same concentrations (Figure 5b). The apparent dependence on gemcitabine made the RNA-binding protein HuR (a.k.a. ELAVL1) a conspicuous candidate. Indeed, previous studies have shown that in pancreatic cells HuR is involved in post-transcriptionally stabilizing the translation of many important proteins.^60, 61^ Specifically for our context, HuR has been shown to be a key mediator of gemcitabine's efficacy in cancer cells through its stabilization of dCK mRNA.^56^ We thus hypothesized HuR's involvement in mediating the observed behavior. To investigate this hypothesis, we carried out an HuR ribonucleoprotein immunoprecipitation (RIP) assay in MIA PaCa-2 cells. As Figure 5d and Supplementary Figure S6E show, GPRC5A mRNA is bound by HuR, as is dCK (RIP positive control). When we knocked down HuR by siRNA, the response of *GPRC5A* protein expression to the lower concentrations of gemcitabine was quashed (Supplementary Figures S7A and B), particularly in the 40–100 nM regime.

### HuR post-transcriptionally regulates GPRC5A in MIA PaCa-2 cells

Having shown that HuR directly interacts with GPRC5A's mRNA (Figure 5d), we sought to examine the possibility that HuR underlies the changes in GPRC5A protein expression levels that we observed following gemcitabine exposure. We first treated MIA PaCa-2 cells with 400 nM of gemcitabine and quantitated mRNA expression levels at several time points. As Figure 5e shows, early on (from 0 to 18 h), GPRC5A mRNA levels remained unchanged whereas the abundance of GPRC5A protein level increased markedly as a function of time (Figure 5f and Supplementary Figure S7C). During this time interval, even though the *total* amount of available HuR protein *did not change significantly*, HuR protein's cytoplasmic levels *increased* (Figure 5f and Supplementary Figures S7C and D).^60^

Using a RIP assay, we showed that between 0 and 18 h, the interaction between HuR protein and GPRC5A mRNA increased (Figure 5g) – note that the transcriptional abundance of GPRC5A remains unchanged during this time interval (Figure 5e). Thus, we conclude that HuR contributes to the observed increase in *GPRC5A* protein levels through mRNA stabilization. Forty-eight hours following gemcitabine treatment, there is significant upregulation of GPRC5A transcription (Figure 5e) and a concomitant increase in *GPRC5A* protein abundance (Figure 5f and Supplementary Figure S7A) and this occurs in the absence of mRNA stabilization by HuR, as evidenced by the HuR RIP assay of Figure 5g.

### HuR binds at least one location in GPRC5A's 3′-UTR

**Figure 6:**
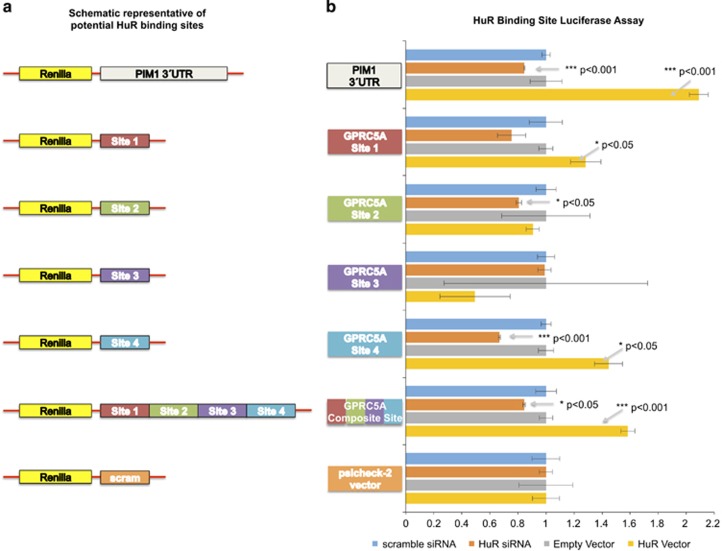
GPRC5A is directly regulated by HuR. (**a**) Schematic representative of potential HuR-binding sites. (**b**) HuR-binding site luciferase assay. Luciferase assay is performed in MIA PaCa-2 cells with psicheck-2 plasmid inserted with predicted HuR-binding sites. PIM1 is used as a positive control.^62^ All numerical data are mean±S.D. *P<0.05, **P<0.01, ***P<0.001, *n*=3

In order to identify HuR's sites of interaction with GPRC5A's mRNA to pursue experimentally, we analyzed previously reported HuR CLIP-seq data and identified four such candidate sites (see Materials and Methods). We cloned separately each of the four sites (labeled 1, 2, 3 and 4). We also cloned the segment containing all four sites labeled as ‘composite site' (Figure 6a). Figure 6b shows the results of luciferase assays using these five constructs. As can be seen, knockdown of HuR by siRNA reduced luciferase signal for the constructs containing sites 2 and 4, and for the ‘composite site' no significant change was observed for the constructs containing sites 1 and 3, respectively. Upregulation of HuR via an HuR overexpression vector increased luciferase signal in the case of sites 1 and 4, and of the ‘composite site' no significant change was observed in the case of sites 2 and 3. PIM1 served as a positive control.^62^

### Knockdown of GPRC5A could induce cell apoptosis, with or without gemcitabine treatment

Our further analyses of GPRC5A on MIA PaCa-2 cells' response to gemcitabine showed that knockdown of GPRC5A enhanced cell apoptosis without gemcitabine treatment; also, that administration of gemcitabine further enhanced pancreatic cancer cell apoptosis (Supplementary Figures S9A and B). At the molecular level, we found that knockdown of GPRC5A inhibited caspase 3 activation and increased accumulation of *γ*H2AX, a marker of DNA damage (Supplementary Figures S9C and D). Gemcitabine treatment could induce caspase 3 activation, as well as γH2AX accumulation in the control group. In the si-GPRC5A-treated group, gemcitabine failed to activate caspase 3 but still resulted in *γ*H2AX accumulation.

## Discussion

As the expression levels of GPRC5A in normal tissues vary very widely across tissues (from highly expressed in lung and colon to lowly expressed lines and pancreas),^4^ it is reasonable to posit that GPRC5A may be playing different roles in each of these contexts. This is further corroborated by previous reports of GPRC5A acting as a tumor suppressor in lung^24^ whereas in, for example, stomach and liver, increased GPRC5A levels are associated with poor prognosis in patients with gastric cancer or hepatocellular carcinoma, respectively.^20, 26^ In pancreas, previous research showed that GPRC5A was upregulated in the cancer state,^6^ but studies of its roles in pancreatic cancer have been limited.

In this report, we present evidence that in multiple pancreatic cancer cell lines the levels of GPRC5A mRNA and protein are higher compared with the normal pancreatic epithelial cell line hTERT-HPNE. We also show that in actual pancreatic tumor samples *GPRC5A* protein levels were dramatically higher than in normal pancreatic ductal cells.

When we overexpress GPRC5A in hTERT-HPNE the cells' ability to form colonies and to migrate compared with control is enhanced. Conversely, when we inhibit GPRC5A in pancreatic cancer cell lines such as MIA PaCa-2, Panc-1 and Capan-2 (Figures 3e and h and Supplementary Figures S2E–G and S3A–D) and in normal pancreatic cell line such as hTERT-HPNE (Supplementary Figures 3E–H) we decrease the cells' ability to form colonies and we hinder migration.

Additionally, we are able to establish GPRC5A's participation in the pancreatic cancer cells' response to gemcitabine treatment. Specifically, increasing concentrations of gemcitabine lead to concomitant increases in GPRC5A protein levels. From a temporal standpoint, we show that for the first 18 h following gemcitabine treatment, GPRC5A protein levels increase monotonically but are solely the result of post-transcriptional events. At 48 h following exposure to gemcitabine, we observe a marked increase in GPRC5A mRNA levels. Gemcitabine treatment could induce apoptosis in pancreatic cancer cells (Supplementary Figures S9A and B) that was enhanced when we inhibited GPRC5A with an siRNA (Supplementary Figures S9A and B, Figures 4a–c and Supplementary Figures S4A–H). In addition, our results showed that inhibition of GPRC5A could also sensitize pancreatic cancer cells to Trichostatin A (Supplementary Figure S5C). Additionally, we found that inhibition of STAT3 or NFkB activation could also sensitize pancreatic cancer cells to gemcitabine (Supplementary Figures S9E–I).

In previous studies, we showed that GPRC5A is regulated post-transcriptionally by miR-103a-3p^59^ and, presumably, by other currently unknown miRNAs. In the present study, we present another dimension of post-transcriptional regulation of GPRC5A via its interaction with the RNA-binding protein HuR. Using luciferase assays, we demonstrate that HuR binds at least one site in the 3′-UTR of GPRC5A. Possibly more HuR-binding sites exist elsewhere in GPRC5A's rather long mRNA. We show that following the cellular stress caused by gemcitabine treatment, HuR protein translocates to the cytoplasm where it binds GPRC5A's mRNA and leads to a monotonic increase in GPRC5A protein levels for at least 18 h (Supplementary Figure S7D). By 48 h following gemcitabine exposure, the association of HuR and GPRC5A mRNA returns to background levels and post-transcriptional control of GPRC5A's mRNA decreases. MiR-103a-3p abundance, a known regulator of GPRC5A,^59^ does not change appreciably compared with 18 h (Supplementary Figure S7F). However, cytoplasmic GPRC5A protein levels increase even further at 48 h following gemcitabine exposure, the result of a transcriptional increase of GPRC5A mRNA in the cells.

In addition to gemcitabine, GPRC5A expression is affected following treatment with either 5-FU or Oxaliplatin. Interestingly, 5-FU treatment upregulates both GPRC5A mRNA and protein level at 48 h after administration (Supplementary Figures S8A–C) whereas Oxaliplatin has an effect on GPRC5A mRNA and protein expression as early as 24 h following treatment (Supplementary Figures S8D–F). These findings suggest that other factors may be involved in regulating GPRC5A expression in the presence of chemical stressors.

**Figure 7:**
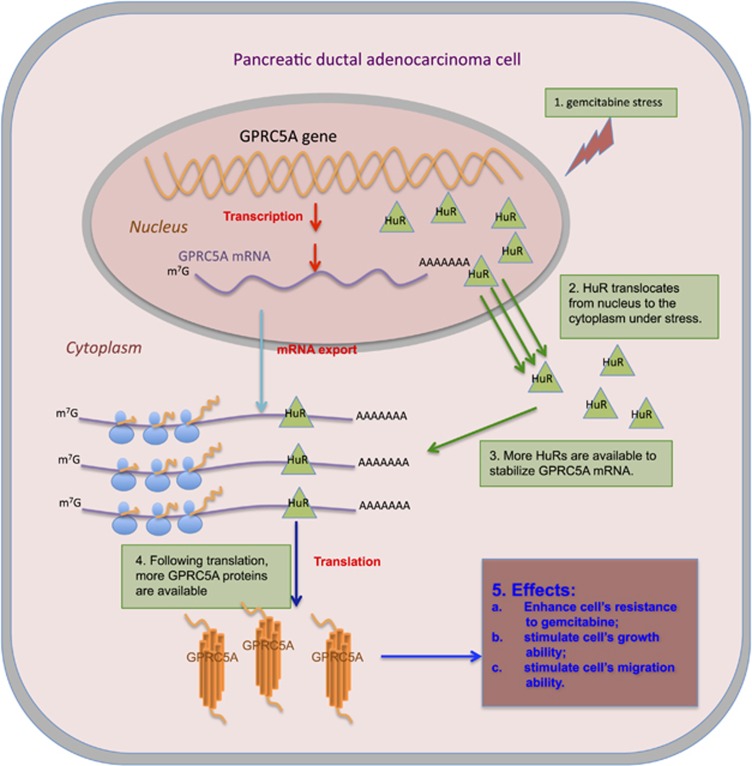
A schematic model for the gemcitabine treatment enhancing GPRC5A expression with the help of HuR in a pancreatic cancer cell. Upon gemcitabine stress (Step 1), HuR molecules translocate from the nucleus to the cytoplasm (Step 2). More HuR molecules are available in the cytoplasm to stabilize GPRC5A mRNA (Step 3). More GPRC5A protein is produced as a result (Step 4). GPRC5A protein enhances the pancreatic cancer cell's resistance to gemcitabine, and stimulates the pancreatic cancer cell's growth ability as well as migration ability (Step 5)

In summary, we presented evidence that suggests that GPRC5A participates in a complex set of interactions in the pancreatic cancer context (Figure 7). We also show that a co-dependent regulation of GPRC5A and HuR is accented in a time-dependent manner when cells are treated with gemcitabine. By potentially exploiting these interactions via targeting of GPRC5A mRNA we can *increase* the death rate of pancreatic cancer cells following treatment by gemcitabine or Trichostatin A. The increased abundance of GPRC5A in human cancer cell lines, TCGA cancer samples, and pancreatic cancer primary tumors and metastases, and the additional evidence we presented above, namely GPRC5A's interactions with HuR and gemcitabine as well as other potential chemo-drugs, suggest a possible pro-oncogenic role for this gene. Further studies of GPRC5A are warranted as it could prove to be a new candidate target for developing an alternative strategy for treating pancreatic cancer.

## Materials and Methods

### Cell culture

The MIA PaCa-2, hTERT-HPNE, PL-5, PL-45, Capan-2 and Panc-1 cell lines were obtained from American Type Culture Collection (Rockville, MD, USA). The gemcitabine-resistant PL-5-GEM R line was generated in the Brody laboratory (Jimbo *et al.*, unpublished). All cells, except PL-5-GEM R, were grown in DMEM medium (Fisher Scientific, Pittsburgh, PA, USA) supplemented with 10% fetal bovine serum (Life Technologies, Carlsbad, CA, USA), 1% penicillin and streptomycin (Fisher Scientific), and 1% glutamine (Fisher Scientific) at 37 °C in a humidified atmosphere containing 5% CO_2_. PL-5 GEM R cells were grown in the above conditions with a supplement of 400 *μ*M gemcitabine in the medium.

### Cell transfection

The cells were transfected with 50 nM GPRC5A siRNA (Dharmacon, Lafayette, CO, USA) or 50 nM HuR siRNA (Ambion, Austin, TX, USA) by the reverse transfection method using the Lipofectamine RNAiMAX transfection reagent (Life Technologies). Cells transfected with only a scrambled sequence control siRNA (Dharmacon) were examined in parallel as controls. Cells were then subjected to further assays or to RNA/protein extraction after 2 days. Lipofectamine 2000 (Life Technologies) was used for transfection of the psiCHECK-2 reporter vector (Promega, Madison, WI, USA) and pcDNA-3.1 overexpression vector (Life Technologies) and for co-transfection of vectors and siRNAs.

### Colony formation assay

Cells were transfected with either GPRC5A siRNA or scramble siRNA. Twenty-four hours after transfection, cells were split and seeded in six-well plates at low density (~1000 cells per well) and treated with different doses of gemcitabine. The cells were cultured for 6 days at 37 °C in a humidified atmosphere containing 5% CO_2_. The plates were then washed with PBS and stained with crystal violet. The images of each well were scanned. The number of clones re-generated (colonies >50 cells each) was scored to determine the efficiency of clone formation.

### *In vitro* cell invasion assay

The invasiveness of pancreatic cells was evaluated in 24-well Transwell chambers (Corning Inc, Corning, NY, USA), as directed by the manufacturer. Briefly, the upper and lower culture compartments of each well were separated by polycarbonate membranes (8-*μ*m pore size), 300 *μ*l of serum-free DMEM medium were placed into the upper compartment of wells and 600 *μ*l of DMEM medium (Fisher Scientific) supplemented with 10% fetal bovine serum (Life Technologies) were placed into the lower compartment. The Transwell chambers were incubated for 14 h at 37 °C in a humidified atmosphere containing 5% CO_2_. Cell penetration through the membrane was detected by staining the cells on the porous membrane with crystal violet staining and quantified by counting the numbers of cells that penetrated the membrane in five microscopic fields (at × 200 magnification) per filter. Each experiment was carried out in triplicate.

### Cell proliferation assay

Cell proliferation was measured with CCK-8 assay. Cells were transfected with either GPRC5A siRNA or scramble siRNA, or untreated. Twenty-four hours after transfection, cells were split and seeded in 96-well plates at low density (~1000 cells per well) and treated with different doses of gemcitabine, other drugs, or combinations of gemcitabine and Stattic (Selleckchem, Houston, TX, USA) or QNZ (Selleckchem). The cells were cultured for 6 days at 37 °C in a humidified atmosphere containing 5% CO_2_. Then the cells were incubated with 100 *μ*l of 1 × CCK-8 solution for 2 h (Sigma-Aldrich). The absorbance was measured at 490 nm by using Synergy 2 Multi-Mode Microplate Reader (BioTek, Winooski, VT, USA). Data represent the average value of three wells in one experiment. Each experiment was carried out in triplicate.

### Soft agar colony formation assay

Assays of colony formation in soft agar were carried out using standard methods. Briefly, 1.5 ml bottom layers consisting of 0.5% agar medium were prepared in six-well plates by combining one volume of 5% noble agar (Difco Laboratories Inc, Detroit, MI, USA) with nine volumes of DMEM medium with 10% FBS. Cells were trypsinized, centrifuged and resuspended in 0.3% agar medium (1 volume of 3% noble agar and 9 volumes of DMEM medium with 10% FBS). 1 × 10^4^ cells were then plated onto the previously prepared bottom layers. The cells were incubated at 37 °C in a humidified atmosphere containing 5% CO_2_ for 14 days. The plates were then washed with PBS and stained with crystal violet. The images of each well were scanned and colonies were counted.

### RNA isolation and real-time quantitative polymerase chain reaction analysis

Total RNA was extracted using TRIzol reagent (Life Technologies). For the detection of *GPRC5A* mRNA, first-strand complementary DNA was synthesized from 1000 ng of total RNA in the presence of oligo-dT (12–18) primer (Promega) and MMLV reverse transcriptase according to the manufacturer's instructions (Promega). Human glyceraldehyde 3-phosphate dehydrogenase RNA was amplified in parallel as an internal control. Real-time qPCR was performed with SYBR Green PCR Master Mix (Life Technologies) and 20ng of templates using a StepOnePlus Real-Time PCR System (Life Technologies). All primer sequences used for *GPRC5A* mRNA detection are listed in Supplementary Table S1 (available Online). PCRs were performed at 95 °C for 5 min, followed by 40 cycles of 95 °C for 15 s and 60 °C for 1 min. ΔCt was calculated by subtracting the Ct of U6 or glyceraldehyde 3-phosphate dehydrogenase mRNA from the Ct of the mRNA of interest. ΔΔCt was then calculated by subtracting the ΔCt of the negative control from the ΔCt of the sample. The fold change in mRNA was calculated according to the equation 2^ΔΔCt^.

### Computational prediction of putative HuR target sites in GPRC5A

We downloaded 12 public HuR CLIP data sets that included two HITS-CLIP and five PAR-CLIP data sets from GSE28859, one PAR-CLIP data set from GSE29779 and four PAR-CLIP data sets from GSE29943. In each case, we mapped the sequenced reads as we described previously.^63, 64^ We note that these data sets were generated using HEK293 cells, and not pancreatic cancer cells. Consequently, and to minimize the likelihood of working with cell-dependent regulatory interactions,^65^ we (a) employed stringent support thresholds; (b) used different thresholds for each data set that were adaptive to the sequencing depth in each case;^63, 64^ and (c) considered only sites of read accumulation in GPRC5A's 3′UTR that were supported by multiple among the 12 data sets.

### DNA vectors

The coding region of the GPRC5A mRNA with either wild type or mutated start codon was amplified by PCR from MIA PaCa-2 cDNA using high fidelity AccuPrime Taq DNA Polymerase (Life Technologies). The fragments were inserted into the pcDNA-3.1 vector between the *Nhe*I and *Not*I sites. The vectors were labeled GPRC5A WT-CDS or GPRC5A MT-CDS, respectively. The HuR overexpression vector was contributed by Dr. Jonathan Brody.

### Reporter vectors

The predicted HuR-binding sites were synthesized as sense and antisense oligomers, annealed and cloned into a psiCHECK-2 vector. All primers used for these constructs are listed in Supplementary Table S1. The PIM1 psiCHECK-2 vector was contributed by Dr. Fernando Blanco and Dr. Jonathan Brody.

### Luciferase assay

Each psiCHECK-2 vector containing a reporter construct was co-transfected into MIA PaCa-2 cells with HuR siRNA or HuR overexpression vector by using Lipofectamine 2000 according to the manufacturer's protocol for co-transfection of DNA and siRNAs. In parallel, each psiCHECK-2 vector containing a reporter construct was also co-transfected into MIA PaCa-2 cells with control siRNA or pcDNA-3.1 vector as control. Cells were harvested at 48 gemcitabine after transfection, and the Renilla and Firefly luciferase activities in the cellular lysate were assayed by using the Dual-Glo Luciferase Assay (Promega) according to the manufacturer's protocol. Light intensity for each sample was measured by using the Synergy 2 Multi-Mode Microplate Reader (BioTek), and each value from Renilla luciferase was normalized by Firefly luciferase.

### Western blots

Transfected cells were lysed on ice in RIPA lysis buffer (Thermo Scientific, Waltham, MA, USA) containing 1 × complete protease inhibitor (Roche, Indianapolis, IN, USA). Debris was pelletized by centrifugation at 13 200 r.p.m. for 15 min, and protein concentrations were determined using the Pierce BCA assay (Thermo Scientific). Lysates were heat-denatured at 100 °C for 10 min before separation in 10% sodium dodecyl sulfate-polyacrylamide gels and transferred to nitrocellulose membrane (GE Healthcare, Mickleton, NJ, USA). Membranes were blocked with 5% bovine serum albumin (Sigma-Aldrich) in Tris-buffered saline Tween-20 buffer (10 mM Tris, pH 7.6, 150 mM NaCl and 0.1% Tween-20) and probed with primary antibody in Tris-buffered saline Tween-20 with 5% bovine serum albumin at the recommended dilutions at 4 °C. Primary antibodies included GPRC5A antibody (Sigma-Aldrich), beta-actin antibody (Cell Signaling Technology, Beverly, MA, USA), HuR antibody (Santa Cruz Biotechnology, Dallas, TX, USA) and LSD1 antibody (Cell Signaling Technology). Membranes were incubated with secondary antibody (Cell Signaling Technology) diluted in Tris-buffered saline Tween-20 with 5% bovine serum albumin for 1 gemcitabine at room temperature. The signal was detected with Pierce ECL Western Blotting Substrate (Thermo Scientific) and GE ImageQuant LAS 4000 (GE Healthcare).

### RIP analysis

6.5 × 10^6^ MIA PaCa-2 cells were plated to ~65% confluency in 100 mm dish (Corning Inc., Corning, NY, USA). Cells were treated with 400 nM gemcitabine (Selleckchem) for 0, 2, 18 and 48 h. Cells were then trypsinized and washed with DPBS (Corning Inc.). After lysis, immunoprecipitation was performed using anti-HuR and IgG antibodies (MBL International, Woburn, MA, USA) as described in the product manuscript. The RNA was quantified by RT-qPCR using an ABI StepOnePlus qPCR System. GAPDH and dCK mRNAs were included as a loading control.^60^

### Statistical analysis

Statistical analysis was performed using the Excel (Microsoft, Redmond, WA, USA), SPSS (IBM, Armonk, NY, USA) and GraphPad Prism (La Jolla, CA, USA). Unless otherwise indicated, the level of significance for the difference between dose response data sets was assessed using two-way ANOVA. Other data sets were assessed using *t-*test. Data are expressed as the means±S.D. *P*-value≤0.05 was considered statistically significant.

### Immunohistochemical analysis

The immunohistochemistry assay was performed following the standard protocol. Specifically, the antigen was retrieved with citrate buffer, pH 6.0 at 98 °C for 20 min. The GPRC5A antibody HPA007928 (Sigma-Aldrich) was used in 1:300 dilution and incubated for 30 min. The stained tissue microarrays (TMA) were analyzed in a blind manner by a pathologist (LB). Each TMA core was given a score from 0 to 3 based on the GPRC5A staining intensity of ductal cells: 0 represents no staining of ductal cells, 1 represents weak staining of ductal cells, 2 represents medium staining of ductal cells, 3 represents strong staining of ductal cells. Additionally, for each code the percentage of stained cells was noted.

## Abbreviations

CLIP-seq: cross-linking followed by immunoprecipitation and sequencing
HuR: human antigen R
PCRs: polymerase chain reaction
PDAC: pancreatic ductal adenocarcinoma
PL-5-GEM R: gemcitabine-resistant PL-5 cell line
*RAI3*: retinoic acid-induced gene 3
TCGA: The Cancer Genome Atlas
TMA: tissue microarrays
UTR: untranslated region
dCK: deoxycytidine kinase
mRNA: messenger RNA
miRNAs: microRNAs
ncRNAs: non-coding RNAs
qPCR: quantitative polymerase chain reaction

## Supplementary Material

Supplementary Informations
