# Supplementary material for: GPRC5A is a potential oncogene in pancreatic ductal adenocarcinoma cells that is upregulated by gemcitabine with help from HuR
Source: Cell Death Dis. 2016 Jul 14;7(7):e2294–. doi: 10.1038/cddis.2016.169 (PMC4973341; doi:10.1038/cddis.2016.169)
Supplement: Supplementary Informations [file cddis2016169x1.pdf]

**SUPPLEMENTAL INFORMATION**

**Supplemental Figure 1.** Immunohistochemistry staining of GPRC5A in normal pancreatic tissues, primary PDAC samples and metastases, chronic pancreatitis, islet cell tumors and its metastases.

**Supplemental Figure 2.** Abnormal expression of GPRC5A protein has an impact on pancreatic cells' colony formation ability and migration ability. A. Quantification of Figure 2E. B. Quantification of Figure 3B. C. Quantification of Figure 3C. D. Quantification of Figure 3D. E. Quantification of Figure 3F. F. Quantification of Figure 3G. G. Quantification of Figure 3H. All numerical data are mean ± sd. * $P<0.05$, ** $P<0.01$, *** $P<0.001$, n=3.

**Supplemental Figure 3.** Knockdown of GPRC5A by siRNA reduces Panc-1, Capan-2 and hTERT-HPNE cells' migration or colony formation ability. A. GPRC5A protein expression in Panc-1 cells after treatment with siRNA. B. Cell migration assay performed with Panc-1 cells after siRNA treatment. C. GPRC5A protein expression in Capan-2 cells after treatment with siRNA. D. Cell migration assay performed with Capan-2 cells after siRNA treatment. E. GPRC5A mRNA expression in hTERT-HPNE cells after treatment with siRNA. F. GPRC5A protein expression in hTERT-HPNE cells after treatment with siRNA. G. Soft agar colony formation assay performed with hTERT-HPNE cells after siRNA treatment. H. Quantification of Supplemental Figure 3G. All numerical data are mean ± sd. * $P<0.05$, ** $P<0.01$, *** $P<0.001$, n=3.

**Supplemental Figure 4** Knockdown of GPRC5A enhances pancreatic cancer cells' death in face of gemcitabine treatment. A. Panc-1 cells' response to different concentrations of gemcitabine treatment. B. Knockdown of GPRC5A in Panc-1 cells enhanced cells' death when treated with gemcitabine. C. Knockdown of GPRC5A in Panc-1 cells reduced cells' colony formation ability when treated with gemcitabine. D. PL-5 cells' response to different concentrations of gemcitabine treatment. E. Knockdown of GPRC5A in PL-5 cells enhanced cells' death when treated with gemcitabine. F. Knockdown of GPRC5A in PL-5 cells reduced cells' colony formation ability when treated with gemcitabine. G. Knockdown of GPRC5A in PL-5 GEM R cells enhanced cells' death when treated with gemcitabine. H. Knockdown of GPRC5A in PL-5 GEM R

cells reduced cells' colony formation ability when treated with gemcitabine. All numerical data are mean ± sd. * P<0.05, ** P<0.01, *** P<0.001, n=3.

**Supplemental Figure 5** MIA PaCa-2 cells' response to different chemo-drugs treatment after knockdown of GPRC5A by siRNA. A. GPRC5A expression in MIA PaCa-2 cells after knockdown of GPRC5A by siRNA. B. MIA PaCa-2 cells' response to 5-FU treatment. C. MIA PaCa-2 cells' response to Trichostatin A treatment. D. MIA PaCa-2 cells' response to Oxaliplatin treatment. E. MIA PaCa-2 cells' response to Olaparib treatment. F. MIA PaCa-2 cells' response to Decitabine treatment. G. MIA PaCa-2 cells' response to Tretinoin treatment. All numerical data are mean ± sd. * P<0.05, ** P<0.01, *** P<0.001, n=3.

**Supplemental Figure 6.** Gemcitabine treatment enhances GPRC5A expression in PL-5 and MIA PaCa-2 cells. A. Quantification of Figure 5A. B. GPRC5A protein expression in PL-5 cells after 48 hrs treatment of gemcitabine in different concentrations. C. Quantification of Supplemental Figure 5B. D. GPRC5A mRNA expression in PL-5 cells after 48 hrs treatment of gemcitabine in different concentrations. E. HuR immunoprecipitation result. All numerical data are mean ± sd. * P<0.05, ** P<0.01, *** P<0.001, n=3.

**Supplemental Figure 7.** Gemcitabine treatment enhances GPRC5A expression in MIA PaCa-2 cells. A. GPRC5A protein expression in MIA PaCa-2 cells after 48 hrs treatment of gemcitabine in different concentrations combined either with scramble siRNA treatment or with HuR siRNA treatment. B. Quantification of Supplemental Figure 7A. C. Quantification of Figure 5F. D. Immunofluorescence staining of HuR post-gemcitabine treatment. E. HuR immunoprecipitation after gemcitabine treatment. F. miR-103a-3p expression in MIA PaCa-2 cells after 400nM gemcitabine treatment for 0 hrs, 2 hrs, 18 hrs and 48 hrs. All numerical data are mean ± sd. * P<0.05, ** P<0.01, *** P<0.001, n=3.

**Supplemental Figure 8.** 5-FU and Oxaliplatin treatment enhances GPRC5A expression in MIA PaCa-2 cells differently over time. A. GPRC5A mRNA expression in MIA PaCa-2 cells after 24 hrs or 48 hrs treatment of 5-FU in different concentrations. B. GPRC5A protein expression in MIA PaCa-2 cells after 24 hrs or 48 hrs treatment of 5-FU in different concentrations. C. Quantification of Supplemental Figure 8B. D. GPRC5A

mRNA expression in MIA PaCa-2 cells after 24 hrs or 48 hrs treatment of Oxaliplatin in different concentrations. E. GPRC5A protein expression in MIA PaCa-2 cells after 24 hrs or 48 hrs treatment of Oxaliplatin in different concentrations. F. Quantification of Supplemental Figure 8E. All numerical data for qRT-PCR are mean ± sd. * P<0.05, ** P<0.01, *** P<0.001, n=3.

**Supplemental Figure 9** Knockdown of GPRC5A by siRNA enhances MIA PaCa-2 cells' apoptosis combined with gemcitabine treatment. A. The percentage of apoptotic cells in different conditions. B. Quantification of apoptotic cells in different conditions. C. Protein expression in MIA PaCa-2 cells after treated with si-GPRC5A and gemcitabine. D. Quantification of protein expression in MIA PaCa-2 cells after treated with si-GPRC5A and gemcitabine. E. MIA PaCa-2 cells' response to gemcitabine in combination of Stattic or QNZ treatment. F. Protein expression in MIA PaCa-2 cells after treated with Stattic. G. Protein expression in MIA PaCa-2 cells after treated with QNZ. H. Quantification of protein expression in MIA PaCa-2 cells after treated with Stattic. I. Quantification of protein expression in MIA PaCa-2 cells after treated with QNZ. All numerical data are mean ± sd. * P<0.05, ** P<0.01, *** P<0.001, n=3.

## SUPPLEMENTAL TABLE CAPTIONS

**Supplemental Table 1.** The sequences of all the primers and predicted HuR binding sites used in experiments.

## SUPPLEMENTAL MATERIALS AND METHODS

**Immunoprecipitation** 6.5 X $10^6$ MIA PaCa-2 cells were plated to ~65% confluency in 100mm dish (Corning Inc, Corning, NY, USA). Cells were treated with 400nM gemcitabine (Selleckchem, Houston, TX, USA) for 0hr or 18hrs. Cells were then trypsinized and washed with DPBS (Corning Inc, Corning, NY, USA). Extra-nuclear portion was extracted by using NE-PER nuclear and cytoplasmic extraction kit (Thermo Scientific, Waltham, MA, USA). Then immunoprecipitation was performed with the extra-nuclear portion using anti-HuR and IgG antibodies (MBL International, Woburn, MA, USA) as described in the product manuscript.

**Immunofluorescence** MIA PaCa-2 cells were treated with 400nM gemcitabine for 0hr or 18hrs. Cells were fixed with 4% paraformaldehyde (PFA), 2% sucrose in PBS for 8 min at room temperature. Cells were then washed three times in PBS, blocked and permeabilized for 2 hrs at room temperature in 1% ovalbumin, 0.2% gelatin from cold-water fish in PBS containing 0.01% saponin. Following block, cells were stained for 2 hrs at room temperature with HuR antibody (Santa Cruz Biotechnology, Dallas, TX), washed three times in PBS and then immunostained with corresponding secondary antibody for 45 min at room temperature. After washing three times in PBS, coverslips were mounted with DAPI solution and used for the conventional confocal imaging with Leica confocal microscope.

**Flow cytometry analysis** Cell apoptosis was measured with APC Annexin V/PI (Becton Dickinson, Franklin Lakes, NJ) staining following flow cytometry analysis. Cells were transfected with either GPRC5A siRNA or scramble siRNA. 24 hrs after transfection, replaced the medium with new culture medium to the original volume and treated cells to induce apoptosis by adding gemcitabine (1 μM) or DMSO and incubated for 24 hrs at 37°C in a humidified atmosphere containing 5% $CO_2$. Cell culture medium was collected into 15 mL tube. Cells were trypsinized and then combined with collected medium in 15mL tube, respectively. Annexin V /PI staining was performed following manufacturer's protocol. Stained cells were analyzed with BD Accuri C6 cytometer (Becton Dickinson, Franklin Lakes, NJ) and data was analyzed with FlowJo software (FlowJo, LLC, Ashland, OR).
