# Supplementary material for: GPRC5A is a potential oncogene in pancreatic ductal adenocarcinoma cells that is upregulated by gemcitabine with help from HuR
Source: Cell Death Dis. 2016 Jul 14;7(7):e2294–. doi: 10.1038/cddis.2016.169 (PMC4973341; doi:10.1038/cddis.2016.169)

Supplemental Figure 1

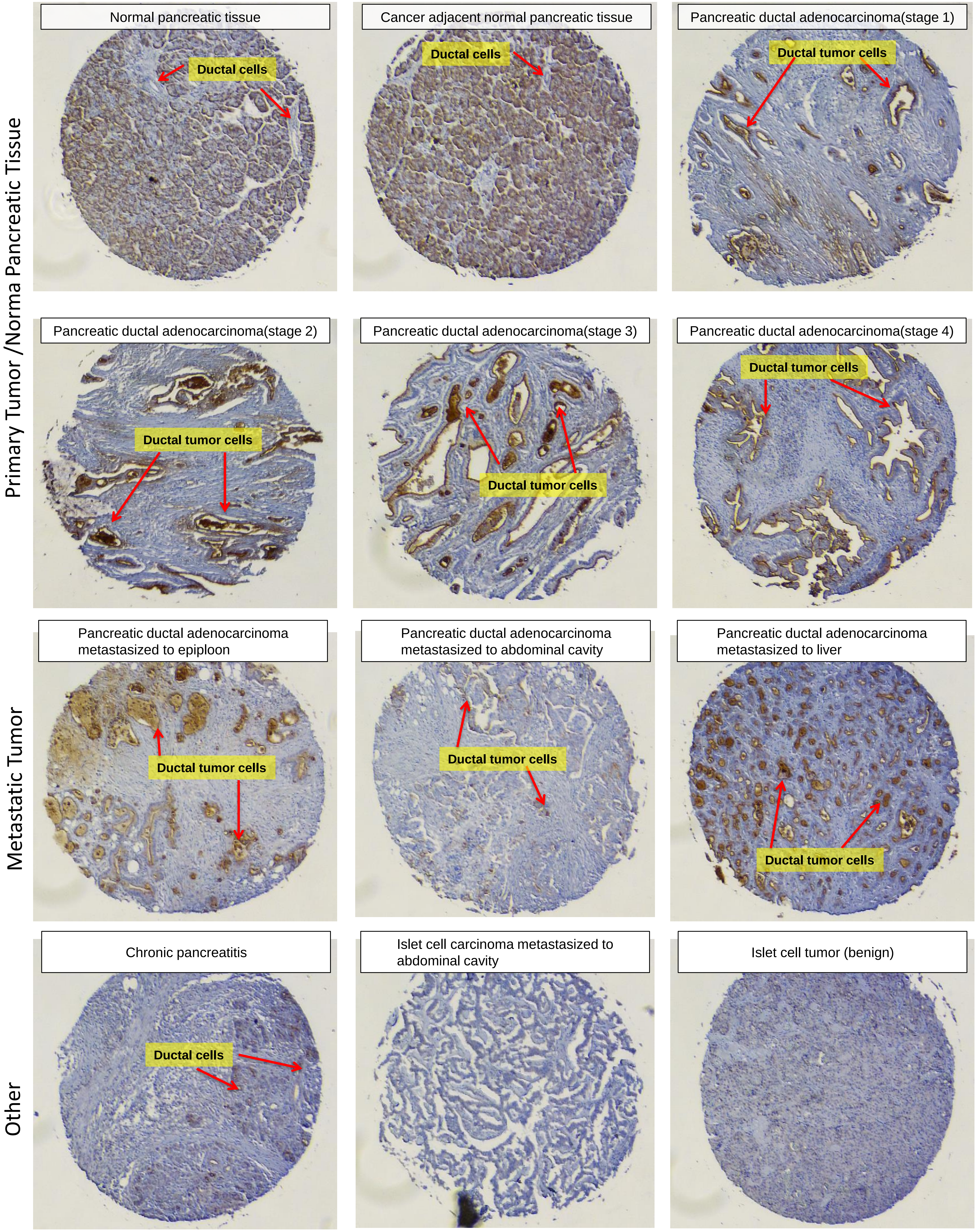

Supplemental Figure 2

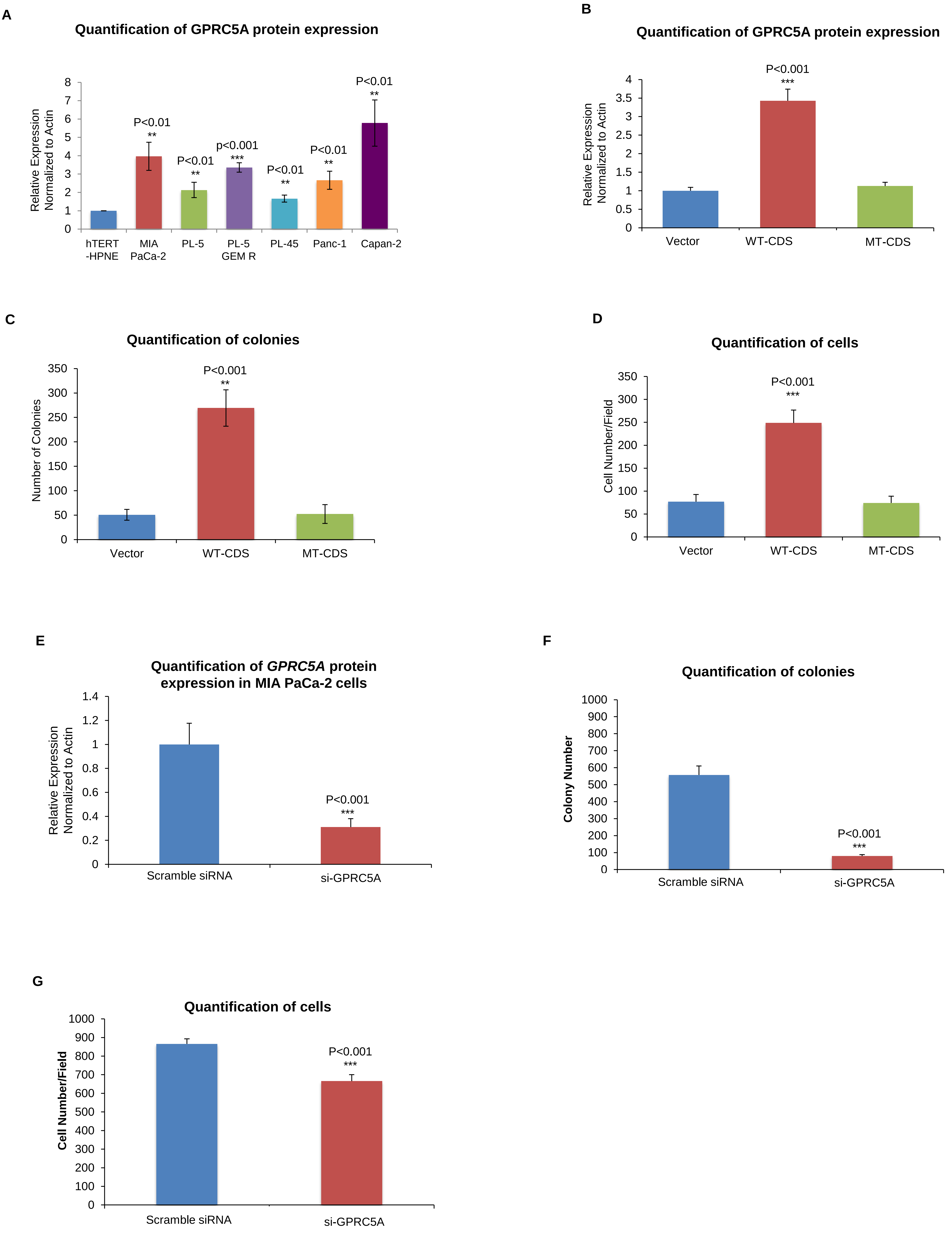

Supplemental Figure 3

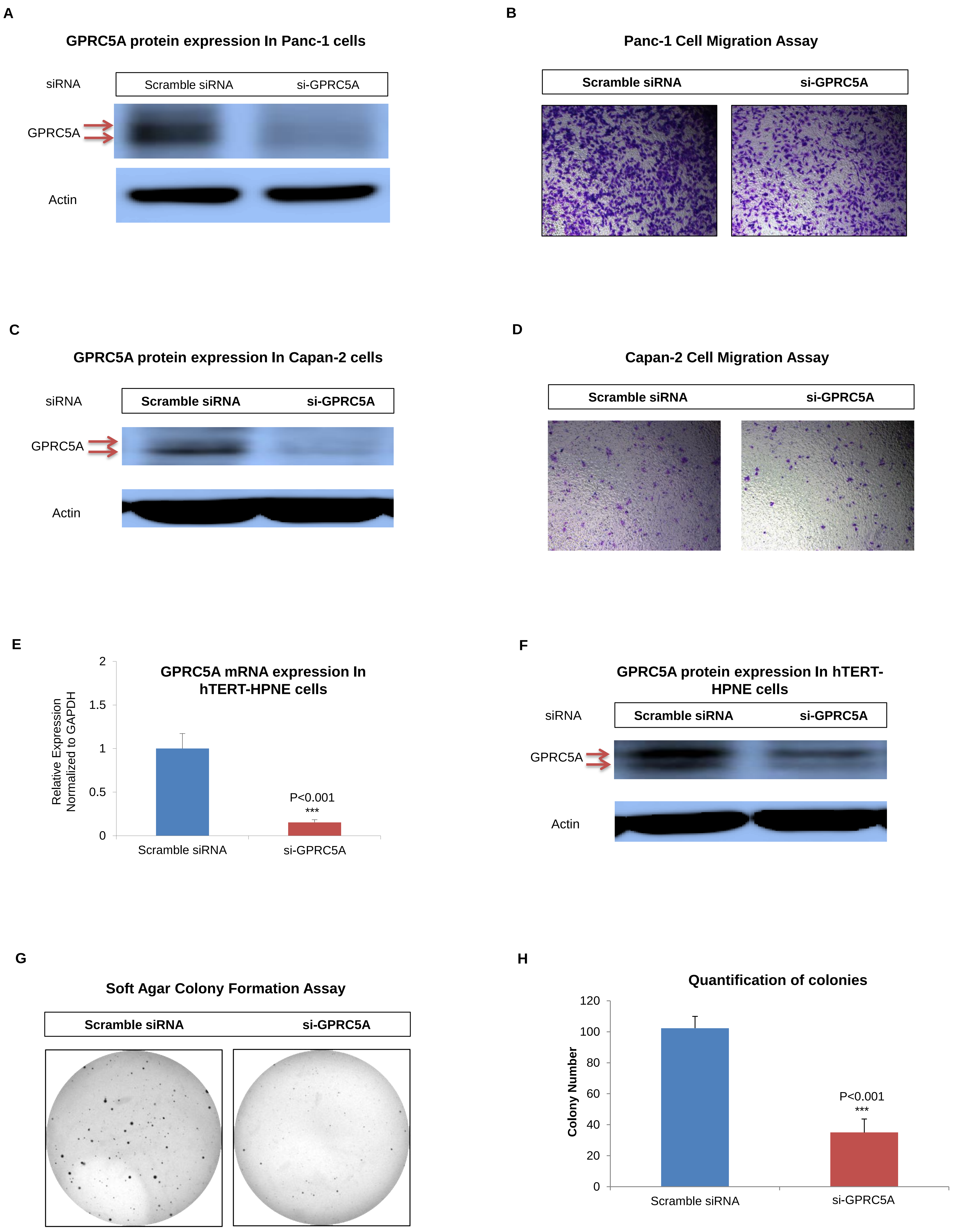

Supplemental Figure 4

A

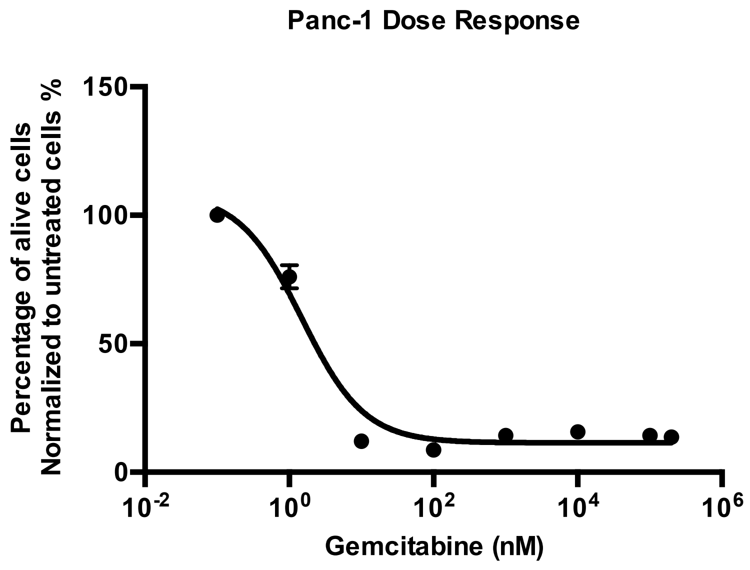

B

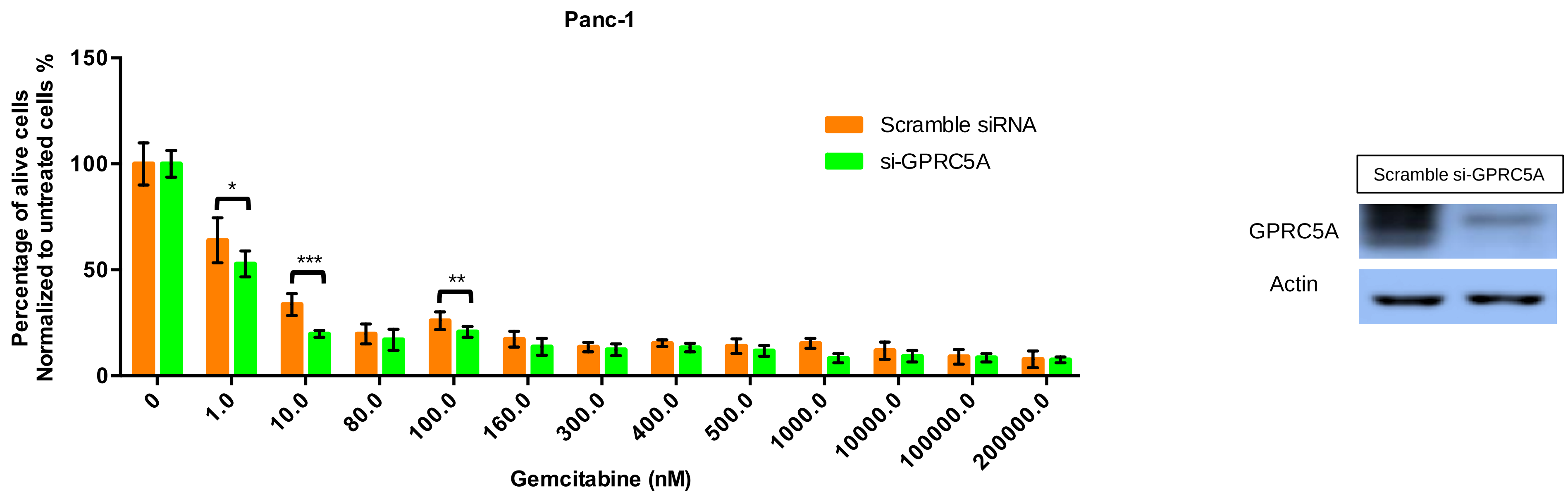

C

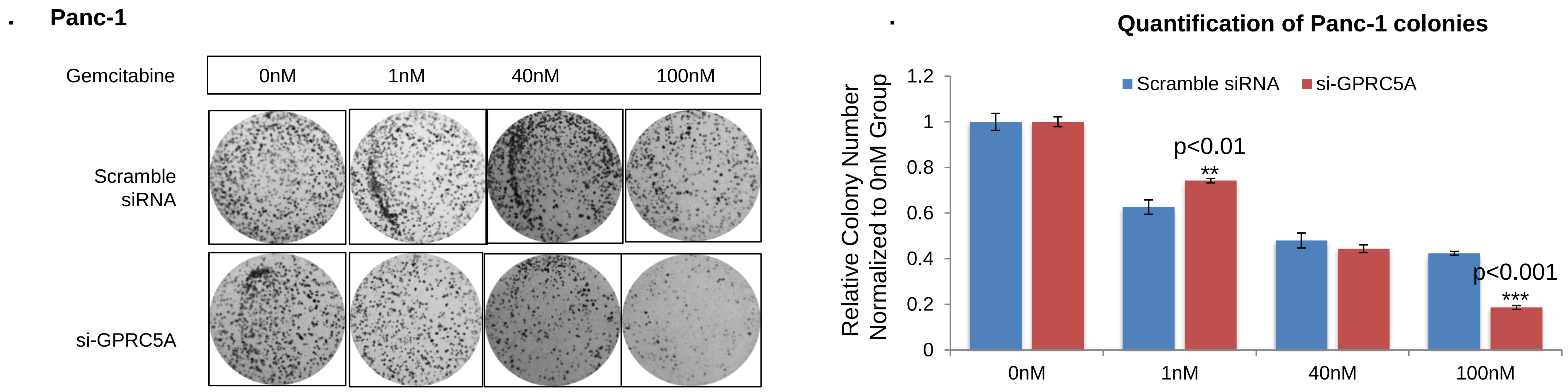

D

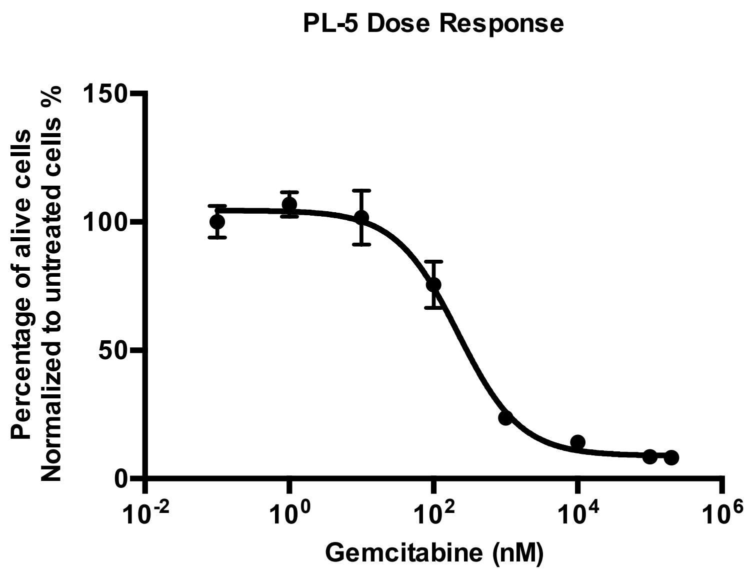

Supplemental Figure 4

E

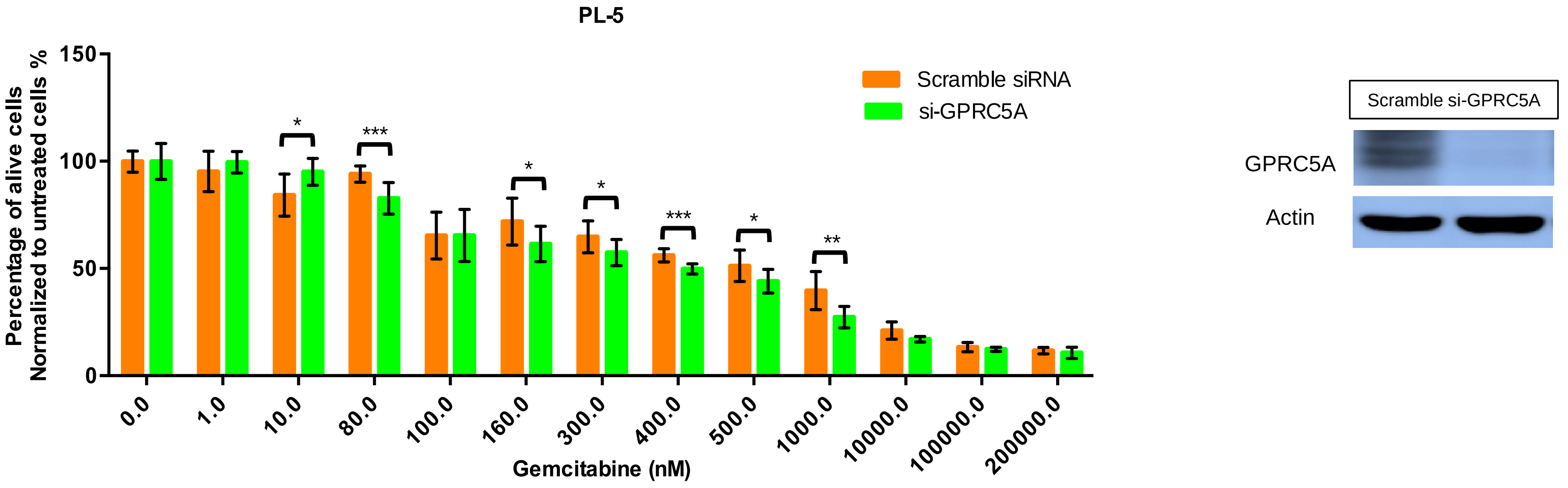

F

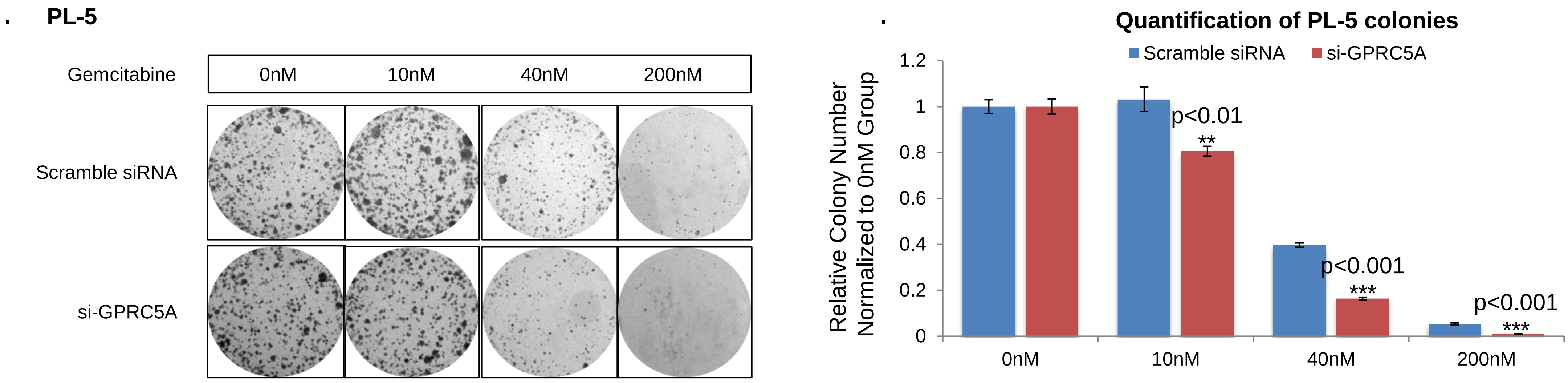

G

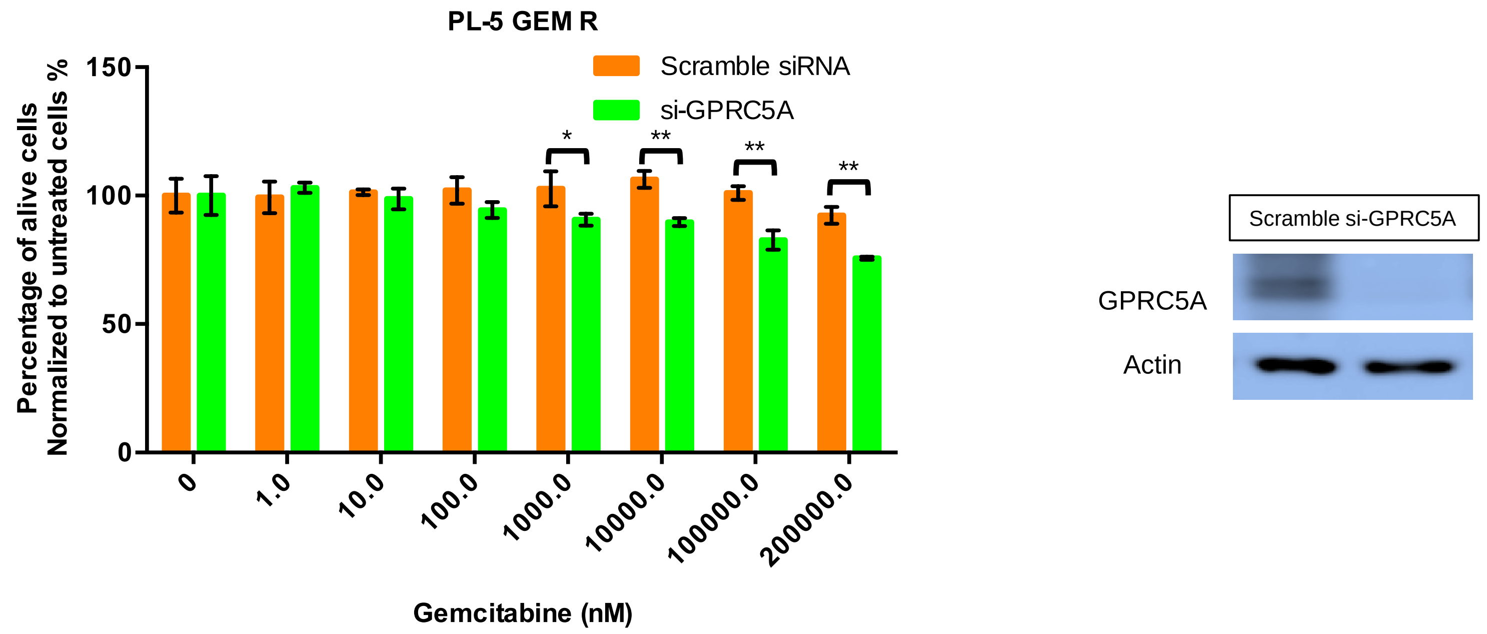

H

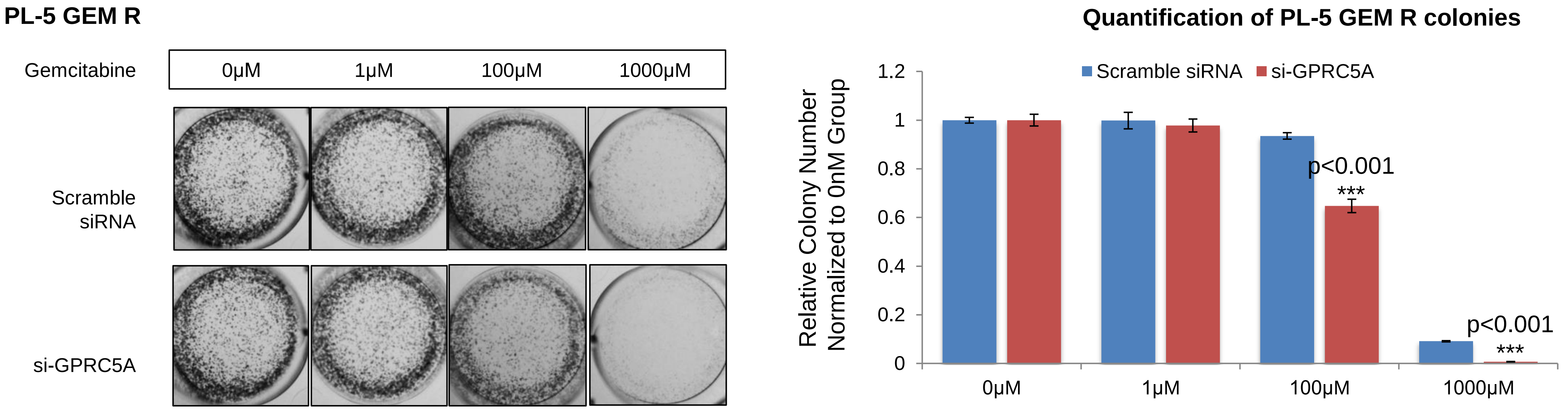

Supplemental Figure 5

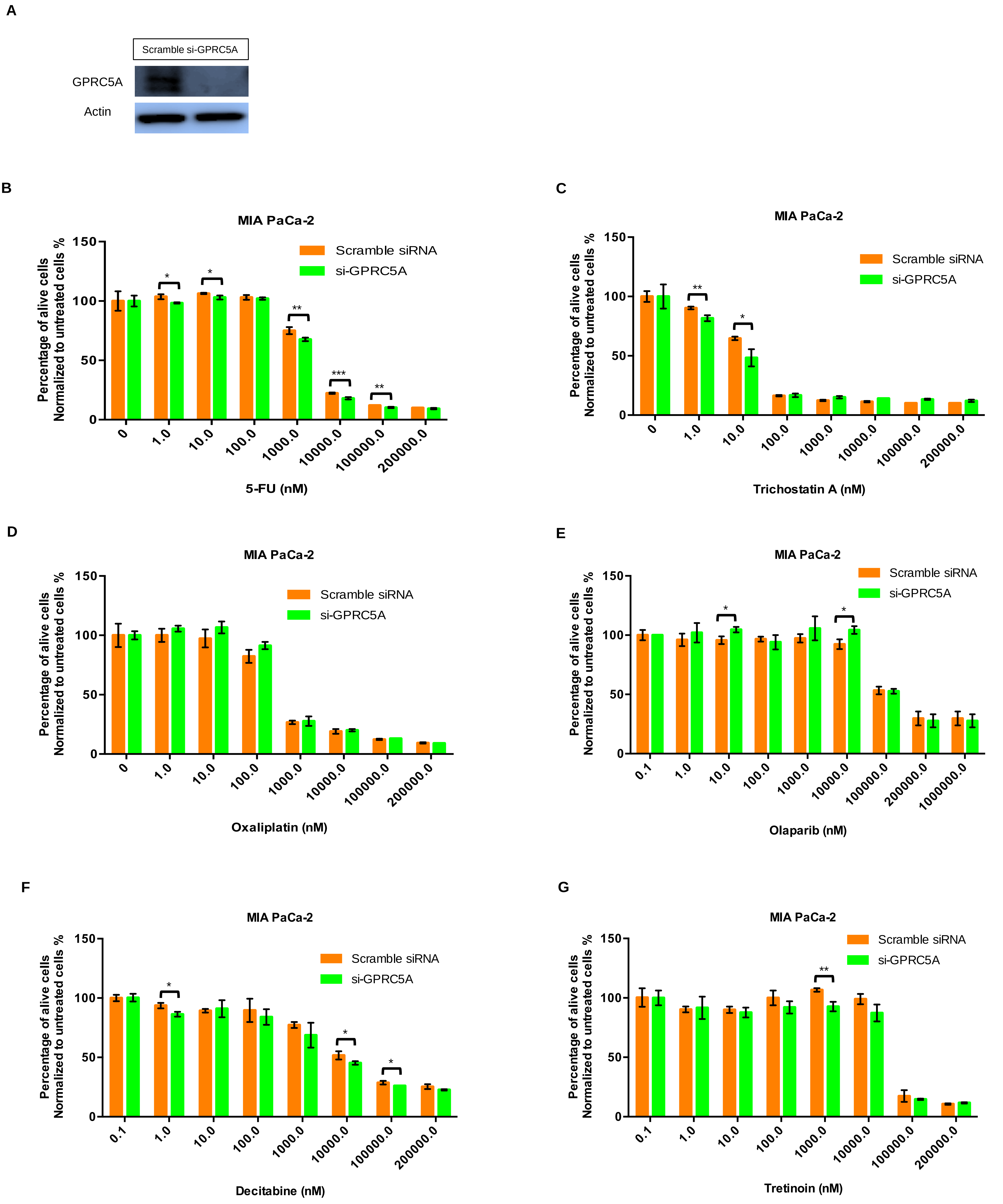

Supplemental Figure 6

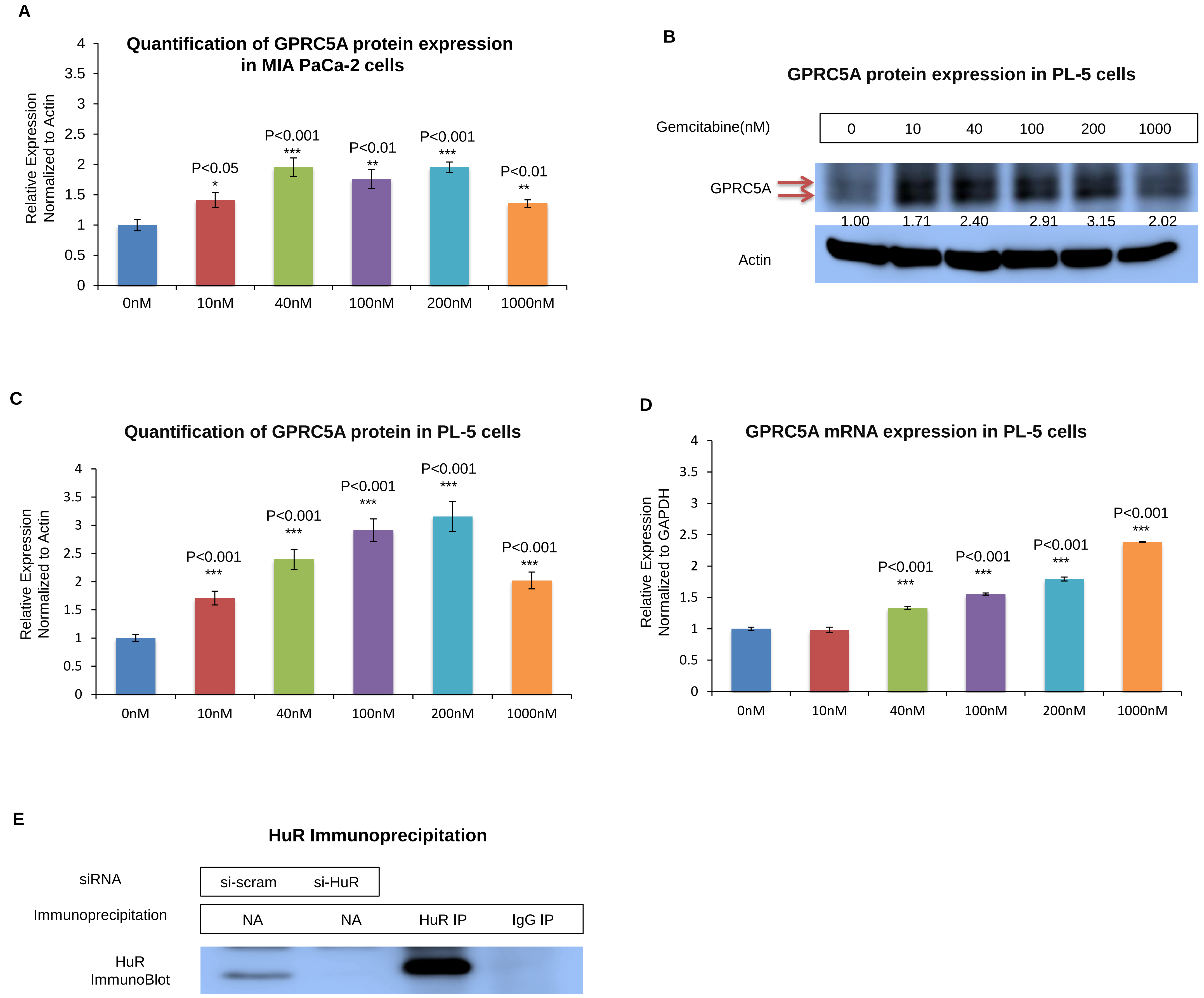

Supplemental Figure 7

A

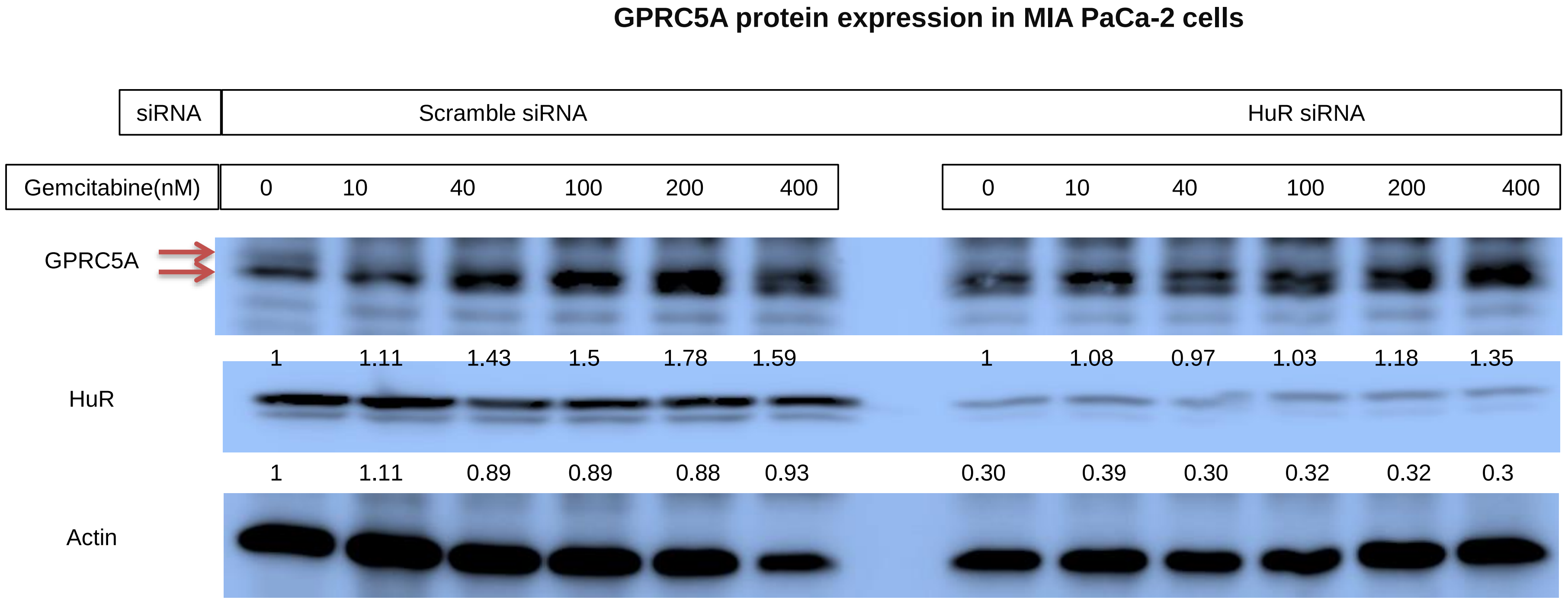

B

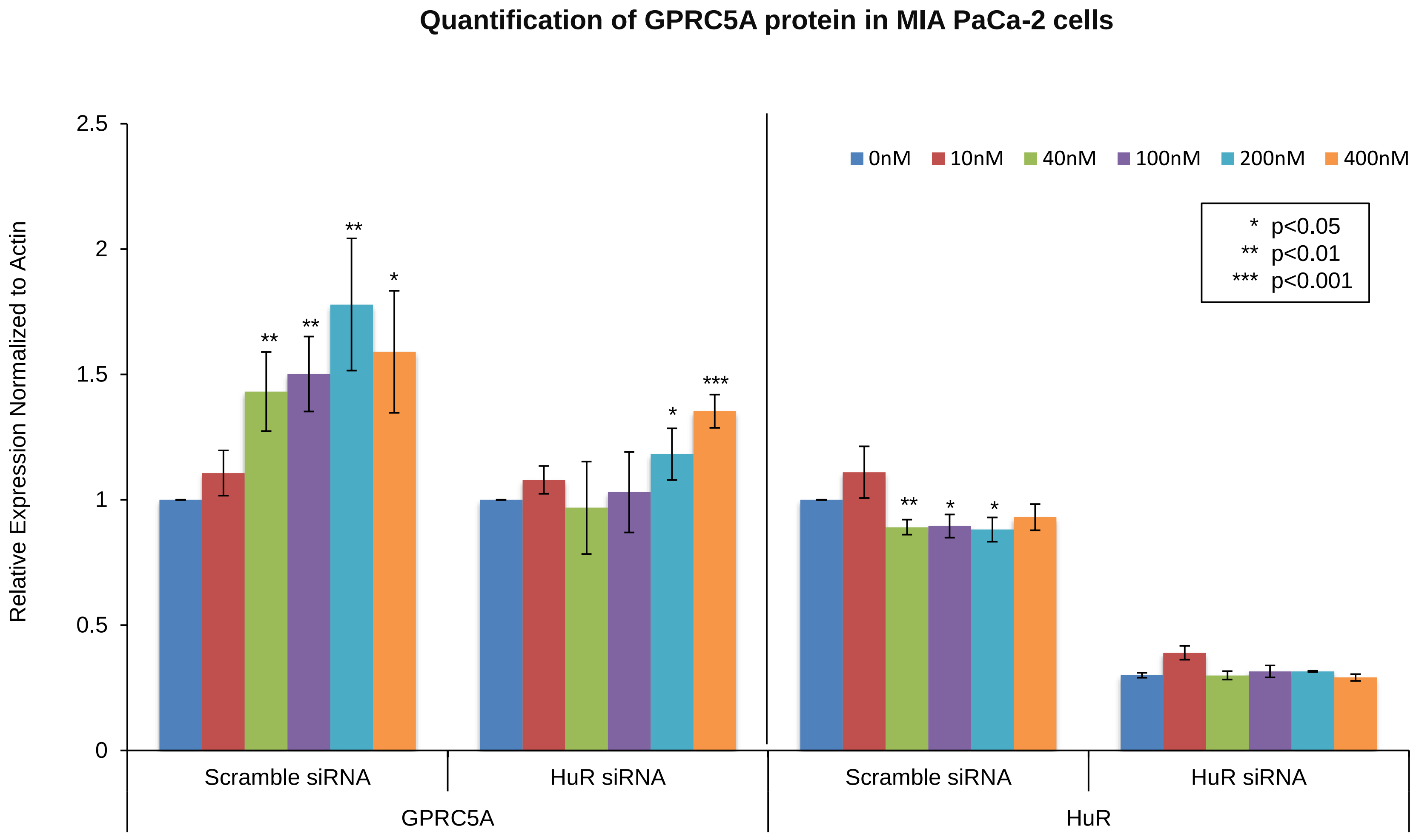

Supplemental Figure 7

C

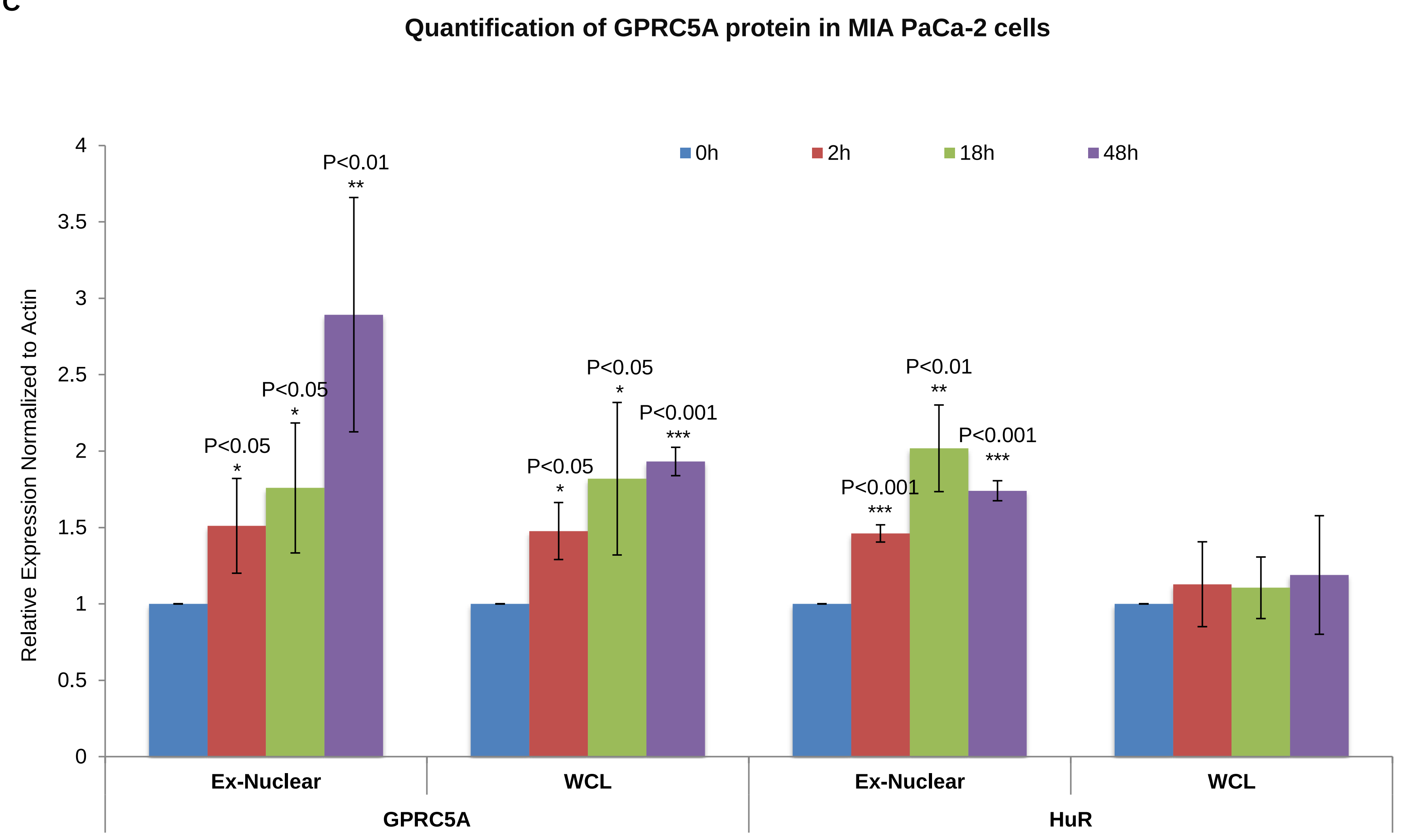

D

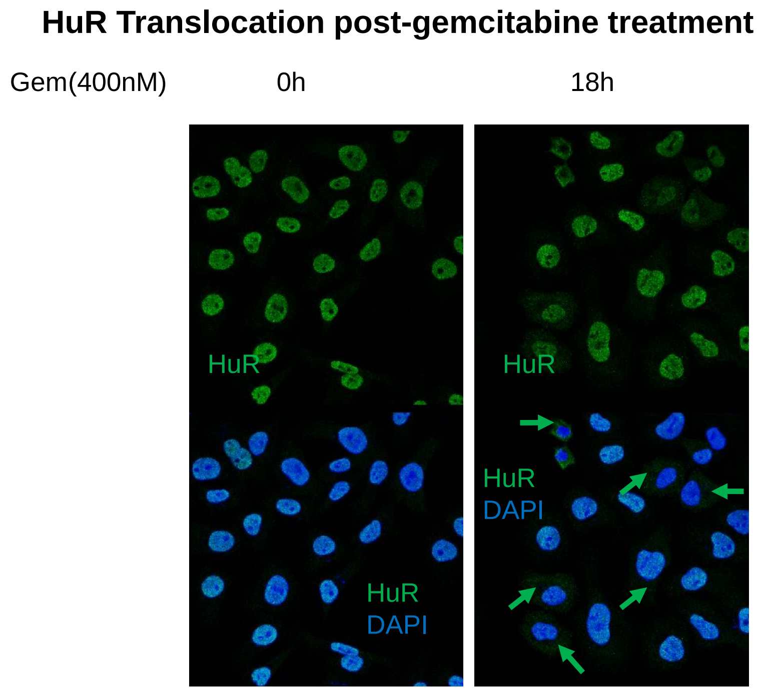

E

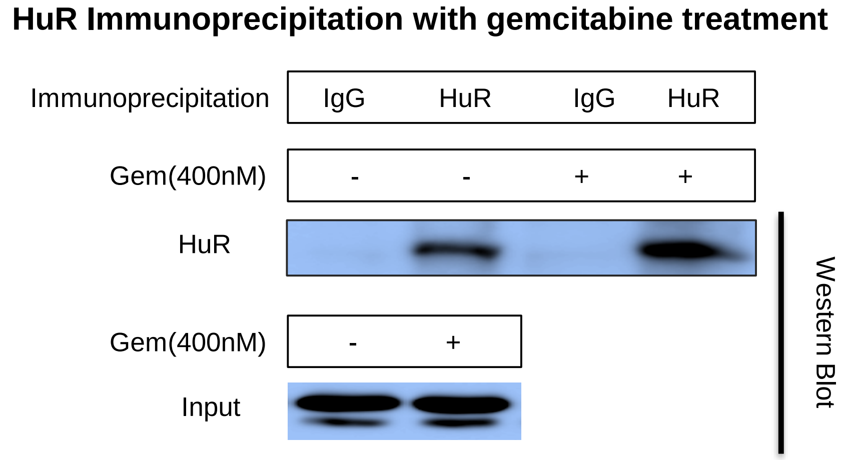

F

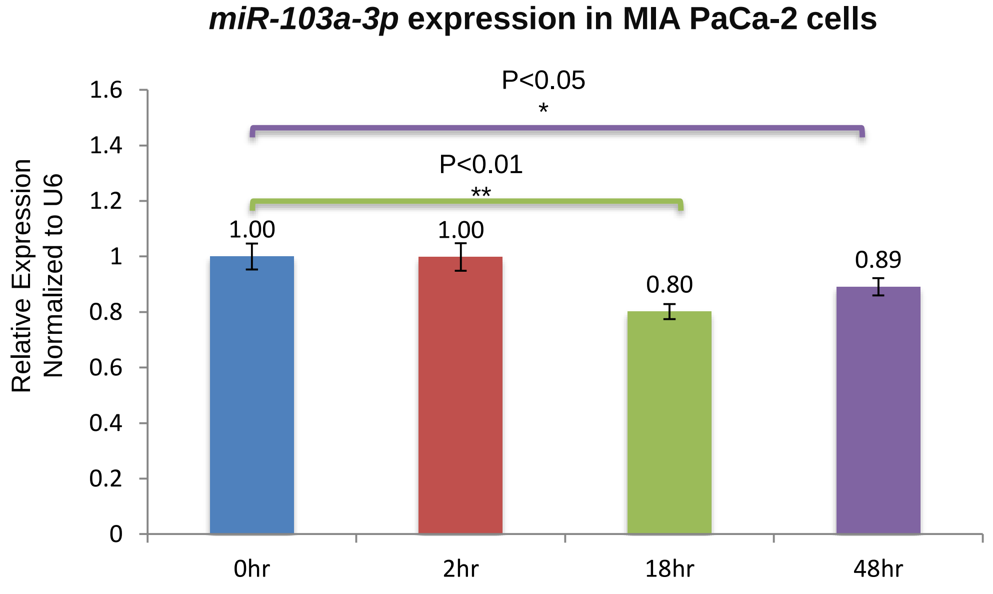

Supplemental Figure 8

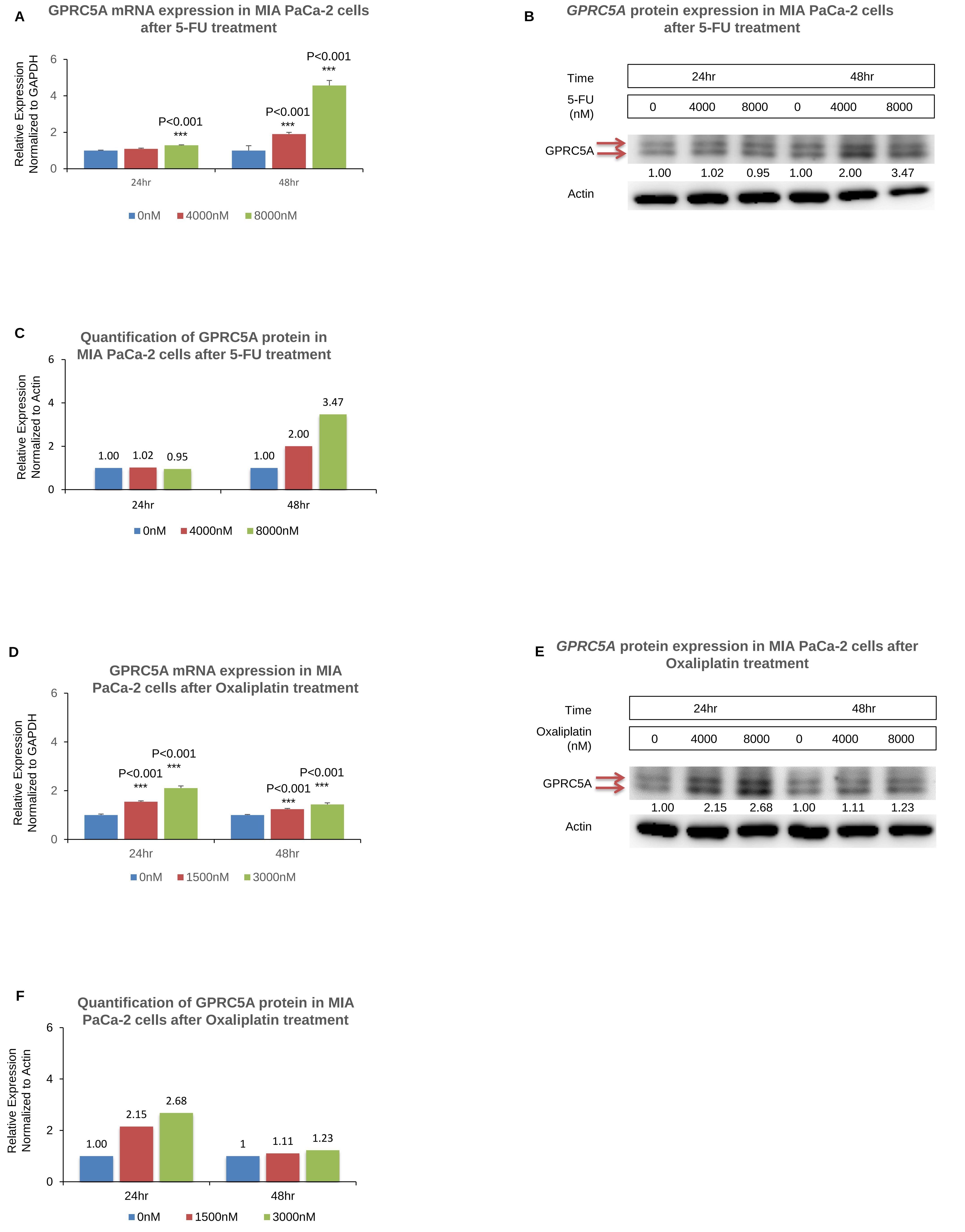

Supplemental Figure 9

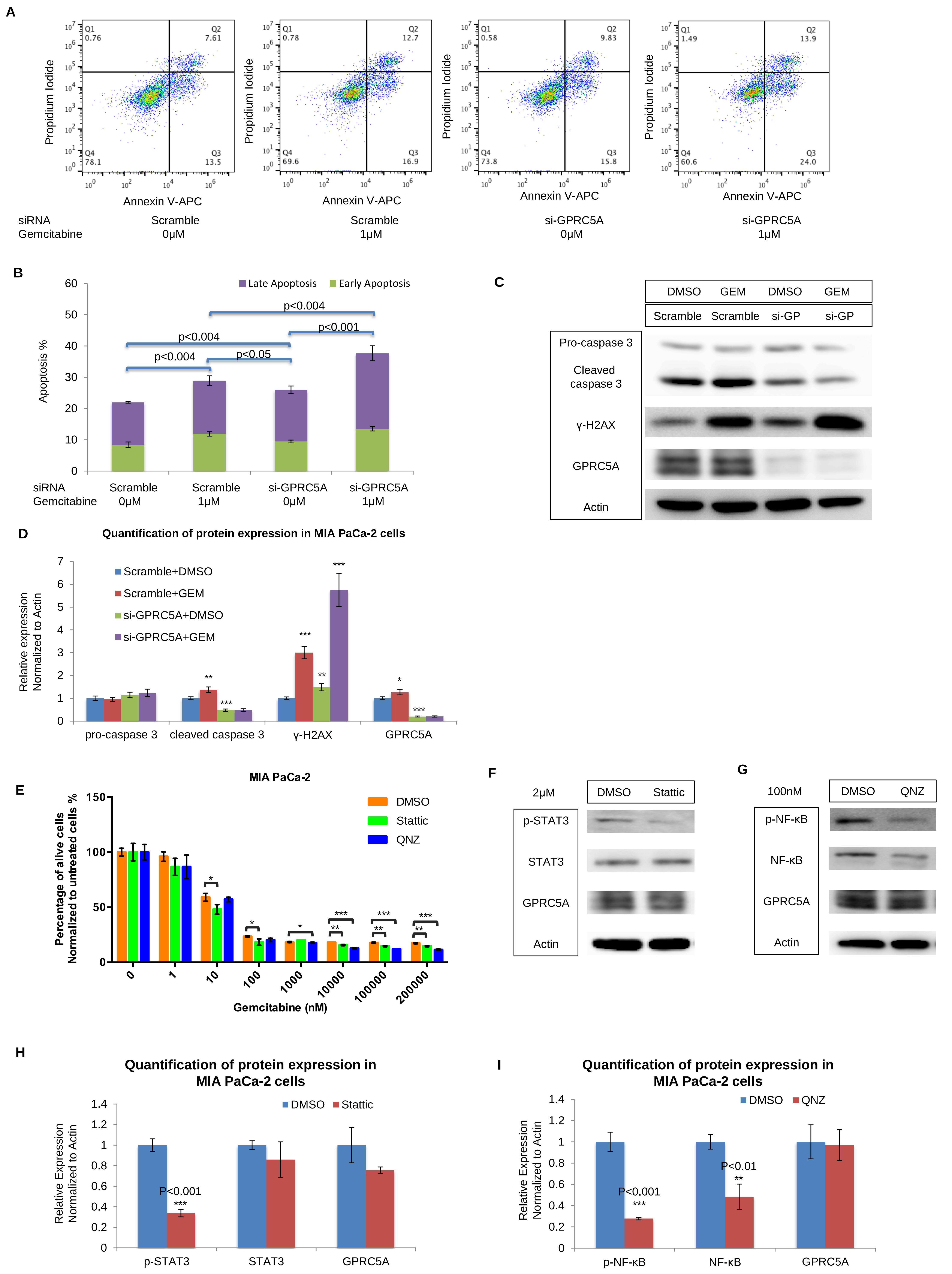

Supplement: Supplementary Figures [file cddis2016169x2.pdf]
