# Supplementary material for: GPRC5A is a potential oncogene in pancreatic ductal adenocarcinoma cells that is upregulated by gemcitabine with help from HuR
Source: Cell Death Dis. 2016 Jul 14;7(7):e2294–. doi: 10.1038/cddis.2016.169 (PMC4973341; doi:10.1038/cddis.2016.169)
Supplement: Supplementary Table 1 [file cddis2016169x3.pdf]

Supplemental Table 1. The sequences of all the primers and predicted HuR binding sites used in experiments.

Primers/Sites	Sequences
GPRC5A-F	GCTGCTCACAAAGCAACGAA
GPRC5A-R	ATAGAGCGTGTCCCCTGTCT
GAPDH-F	GAAAGCCTGCCGGTGACTAA
GAPDH-R	AGGAAAAGCATCACCCGGAG
dCK-F	CTGGCTCCTGCATAGGACAC
dCK-R	CCATTTGGCTGCCTGTAGTC
GPRC5A-WT-CDS-F	CACGGATCCAACGCCTTGGCACTAGGGTCCAGAATGGCTACAACAG
GPRC5A-WT-CDS-R	CAGCTCGAGCTGCCCTCTTTCTTTACTTCATAGTCTTTGTAAGGG
GPRC5A-MT-CDS-F	CACGGATCCAACGCCTTGGCACTAGGGTCCAGAAAGGCTACAACAG
GPRC5A-MT-CDS-R	CAGCTCGAGCTGCCCTCTTTCTTTACTTCATAGTCTTTGTAAGGG
Si-GPRC5A targeting site	TCTTTCAGACCTCACTAGCACA
site3	AGTCTCGAGTGCAGTGGTGCGATCACAGCCCAGTGCAGCCTCGACCACCTGTGCTCAAGCGGCCGCTTT
site4	AGTCTCGAGCCTGTGCTCAAGCAATCCTCCCATCTCCATCTCCCAAAGTGCTGGGATGACAGGGCGGCCGCTTT
site2	AGTCTCGAGCATCCTTTGGATACTTCTTTTAAGTGGGAGTCTCAGGCAACTCAAGTTTAGACCCTTACTCTTTTTGTTTGTTTTTTGAAGCGGCC<br>GCTTT
site1	AGTCTCGAGGACTCCAGTTCTTAGAGGCGCTGTAGTATTTTTTTTTTTTTGTCTCATCCTTTGGATACTTCTTTTAAGTGGGAGTCTCAGCGGCC<br>GCTTT
Composite site	CGGCAGATCTCGAG GGAGCTCAAAGGGATGTGGGCGAAATCTTGAGTCTTCTGAGAAAAC<br>TGTACAAGACACTACGGGAACAGTTTGCCTCCCTCCCAGCCTCAACCACA<br>ATTCTTCCATGCTGGGGCTGATGTGGGCTAGTAAGACTCCAGTTCTTAGA<br>GGCGCTGTAGTATTTTTTTTTTTTTGTCTCATCCTTTGGATACTTCTTTT<br>AAGTGGGAGTCTCAGGCAACTCAAGTTTAGACCCTTACTCTTTTTGTTTG<br>TTTTTTGAAACAGGATCTTGCTCTGTCACCCAGGCTTGAGTGCAGTGGTG<br>CGATCACAGCCCAGTGCAGCCTCGACCACCTGTGCTCAAGCAATCCTCCC ATCTCCATCTCCCAAAGTGCTGGGATGACAGGCGTGA<br>GCGGCCGCTCCCAGCCTAGGC
